# SPRR1A is a key downstream effector of MiR-150 during both maladaptive cardiac remodeling in mice and human cardiac fibroblast activation

**DOI:** 10.1038/s41419-023-05982-y

**Published:** 2023-07-19

**Authors:** Satoshi Kawaguchi, Bruno Moukette, Marisa N. Sepúlveda, Taiki Hayasaka, Tatsuya Aonuma, Angela K. Haskell, Jessica Mah, Suthat Liangpunsakul, Yaoliang Tang, Simon J. Conway, Il-man Kim

**Affiliations:** 1 Department of Anatomy, Cell Biology, and Physiology, Indiana University School of Medicine, Indianapolis, IN USA; 2 Division of Gastroenterology and Hepatology, Indiana University School of Medicine, Indianapolis, IN USA; 3 Vascular Biology Center, Medical College of Georgia, Augusta University, Augusta, GA USA; 4 Herman B Wells Center for Pediatric Research, Indiana University School of Medicine, Indianapolis, IN USA; 5 Krannert Cardiovascular Research Center, Indiana University School of Medicine, Indianapolis, IN USA; 6 Present Address: Department of Emergency Medicine, Asahikawa Medical University, Asahikawa, Hokkaido Japan; 7 Present Address: Internal Medicine Research Unit, Pfizer Inc., Cambridge, MA USA; 8 Present Address: Division of Cardiology, Nephrology, Pulmonology, and Neurology, Department of Internal Medicine, Asahikawa Medical University, Asahikawa, Hokkaido Japan

**Keywords:** Mechanisms of disease, Experimental models of disease, miRNAs, Cell signalling, Heart failure

## Abstract

MicroRNA-150 (miR-150) is conserved between rodents and humans, is significantly downregulated during heart failure (HF), and correlates with patient outcomes. We previously reported that miR-150 is protective during myocardial infarction (MI) in part by decreasing cardiomyocyte (CM) apoptosis and that proapoptotic small proline-rich protein 1a (*Sprr1a*) is a direct CM target of miR-150. We also showed that *Sprr1a* knockdown in mice improves cardiac dysfunction and fibrosis post-MI and that *Sprr1a* is upregulated in pathological mouse cardiac fibroblasts (CFs) from ischemic myocardium. However, the direct functional relationship between miR-150 and SPRR1A during both post-MI remodeling in mice and human CF (HCF) activation was not established. Here, using a novel miR-150 knockout;*Sprr1a*-hypomorphic (*Sprr1a*^*hypo/hypo*^) mouse model, we demonstrate that *Sprr1a* knockdown blunts adverse post-MI effects caused by miR-150 loss. Moreover, HCF studies reveal that *SPRR1A* is upregulated in hypoxia/reoxygenation-treated HCFs and is downregulated in HCFs exposed to the cardioprotective β-blocker carvedilol, which is inversely associated with miR-150 expression. Significantly, we show that the protective roles of miR-150 in HCFs are directly mediated by functional repression of profibrotic *SPRR1A*. These findings delineate a pivotal functional interaction between miR-150 and SPRR1A as a novel regulatory mechanism pertinent to CF activation and ischemic HF.

## Introduction

Controlling microRNA (miRNA or miR) biogenesis in the heart is an important underlying mechanism of heart failure (HF) [1–5]. Intriguingly, novel miR therapies are being investigated in clinical trials for other diseases [6–9] and more recently for HF [10]. We reported that miR-150 is upregulated by the β-blocker carvedilol (Carv), which acts through β-arrestin1-mediated β_1_-adrenergic receptor (β_1_AR) protective signaling [11]. Significantly, using a systemic miR-150 knockout (KO) mouse model, we also showed that miR-150 plays a vital Carv/β_1_AR/β-arrestin1-mediated protective role in myocardial infarction (MI) in part by decreasing cardiomyocyte (CM) apoptosis [12]. More recently, we demonstrated that cardiac-specific miR-150 conditional KO (cKO) mice exhibit enhanced apoptosis and maladaptive post-MI remodeling [13]. Notably, cardiac-specific overexpression of miR-150 attenuated transverse aortic constriction (TAC)-induced cardiac dysfunction [14] whereas miR-150 loss caused a higher degree of cardiac fibrosis after TAC. MiR-150 was also downregulated in cardiac fibroblasts (CFs), not CMs isolated from TAC mice [15]. Moreover, miR-150 inhibited mouse CF activation in vitro [15]. Interestingly, circulating or cardiac miR-150 is downregulated in patients with multiple cardiovascular diseases (CVDs) [16–19] and mouse models of HF [12, 14, 20]. MiR-150 is conserved between rodents and humans and is significantly associated with HF severity and outcome in humans [21]. Collectively, previous studies support the clinical relevance and potential therapeutic application of miR-150 in HF; however, the detailed mechanisms by which miR-150 modulates HF remain elusive.

Small proline-rich protein 1a (SPRR1A) is induced by stress and is highly conserved. SPRR1A is a substrate of transglutaminase (TGase) I/II-catalyzed crosslinking reactions in forming the keratinocyte envelope [22]. We showed that CMs with *Sprr1a* knockdown are protected against apoptosis in the simulated ischemia/reperfusion (sI/R: hypoxia/reoxygenation [H/R]) condition [13]. Adenovirus-mediated ectopic overexpression of *Sprr1a* promoted cardiac fibrosis in vivo after TAC whereas protecting CMs and isolated hearts against 2-deoxyglucose and ex vivo I/R [23]. Moreover, *Sprr1a* overexpression did not affect CM survival after reactive oxygen species treatment or serum deprivation [23]. This prior study indicated both the potential stress-dependent effects of *Sprr1a* and the requirement of in vivo genetic loss-of-function approaches to define the role of *Sprr1a* in the heart. Interestingly, we recently demonstrated that *Sprr1a*-hypomorphic (*Sprr1a*^*hypo/hypo*^) mice are protected against MI [13]. We also reported that *Sprr1a* is upregulated in CMs isolated from mouse hearts post-MI [13], which is consistent with a report on mouse hearts after TAC [23]. Moreover, we showed that left ventricular (LV) *SPRR1A* is upregulated in patients with HF [13] in agreement with mouse studies showing *Sprr1a* upregulation in myocardial injury [24] and renal I/R injury [25]. Notably, we identified proapoptotic *Sprr1a* as a novel direct and functional target of miR-150 in CMs [13]. We also showed that *Sprr1a* is downregulated by Carv in hearts and CMs [13] concurrent with miR-150 upregulation [11]. Given that rodent and human genes of SPRR1A have almost identical genomic organization and exon/intron sizes [26] and have at least one miR-150 binding site, the regulation of SPRR1A by miR-150 and their roles might be conserved. Importantly, we also showed that *Sprr1a* knockdown in mice improves fibrosis post-MI and that *Sprr1a* is upregulated in mouse CFs isolated from ischemic myocardium [13]. A recent proteomic study in CFs also noted that SPRR1A levels are significantly higher in infarct CFs than remote CFs during MI [27]. These previous findings indicate a possible role of *Sprr1a* in CFs; however, whether *Sprr1a* is functionally regulated by miR-150 in HF and human CF (HCF) activation remains unknown.

Using a novel miR-150 KO;*Sprr1a*-hypomorphic (*Sprr1a*^*hypo/hypo*^) mouse model and primary HCFs, we demonstrate here that (i) *Sprr1a* knockdown alleviates cardiac dysfunction, damage, apoptosis, and fibrosis after MI mediated by miR-150 deletion; (ii) *Sprr1a* is upregulated in HCFs subjected to H/R whereas its expression is downregulated in HCFs by Carv, which is inversely associated with the expression of miR-150; and (iii) the protective actions of miR-150 in HCFs are mediated by the functional repression of profibrotic *SPRR1A*. These data directly establish the functional relationship between miR-150 and *Sprr1a* during both post-MI fibrotic remodeling in mice and HCF activation. Our novel findings suggest that profibrotic SPRR1A is a crucial downstream effector of miR-150 in repressing CF activation and maladaptive cardiac remodeling. The miR-150/SPRR1A axis, therefore, may be considered a novel therapeutic target for ameliorating ischemic heart disease and pathological fibrosis.

## Materials and methods

### MiR-150 knockout and hypomorphic *Sprr1a* mutant mice (*Sprr1a*^*hypo/hypo*^) as well as the generation of miR-150 KO;*Sprr1a*^*hypo/hypo*^ mice

Systemic miR-150 KO mice were purchased from the Jackson Laboratory (007750), and their cardiac phenotypes post-MI were reported in our previous study [12]. *Sprr1a*^*hypo/+*^ were obtained from the Mutant Mouse Resource & Research Centers (RRID: MMRRC_049856-UCD). We previously described the detailed methods regarding this mouse line, including the targeting strategy, generation of *Sprr1a*^*hypo/hypo*^ mice, and genotyping strategy [13]. We also reported the cardiac phenotypes of *Sprr1a*^*hypo/hypo*^ mice after MI in a previous study [13]. In the current study, *Sprr1a*^*hypo/hypo*^ mice were bred with miR-150 KO mice to generate the novel miR-150 KO;*Sprr1a*^*hypo/hypo*^ mouse line. All mice were maintained on a C57BL/6J background, and wild-type (WT) littermates were used as controls.

### Ethics committee approval

The animal experiments conducted as a part of this study complied with the Guidelines for the Care and Use of Laboratory Animals published by the US National Institutes of Health. Mice were euthanized by thoracotomy under 1–4% inhaled isoflurane. All experiments with mice were performed according to the protocols approved by the Institutional Animal Care and Use Committee at the Indiana University School of Medicine (approval reference #21189). 8–16 week-old C57BL/6J mice of both sexes were used for this study. Genotype- and sex-matched mice were randomly assigned to experimental groups to mitigate the cage effect. The genotypes of the animals were masked for researchers until the end of the analysis.

### Statistics

**Fig. 1:**
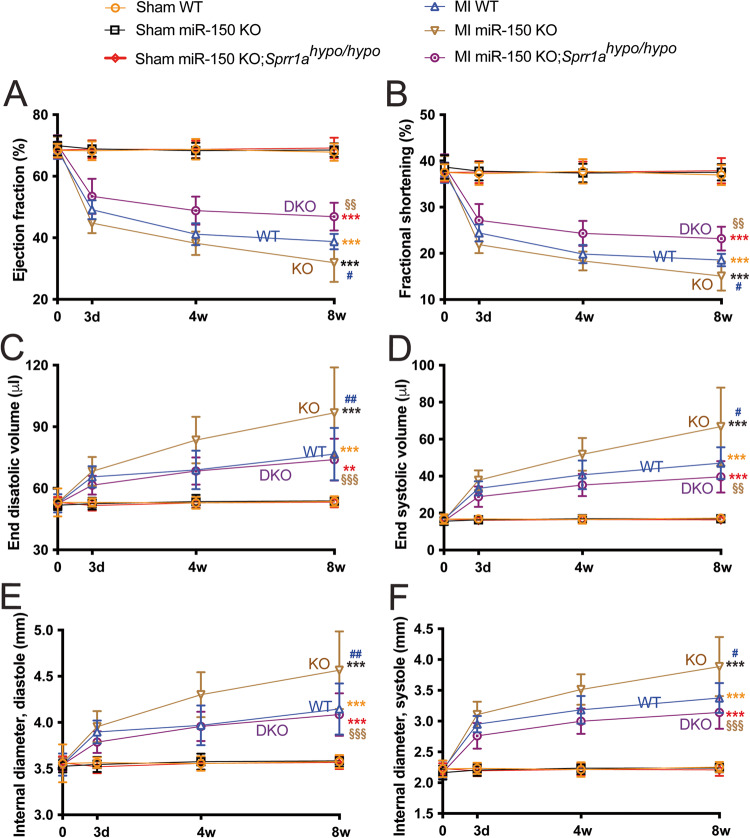
*Sprr1a* knockdown attenuates cardiac dysfunction after myocardial infarction mediated by miR-150 loss. Transthoracic echocardiography was performed on the 6 experimental groups (sham and MI of WT, miR-150 KO, and miR-150 KO;*Sprr1a*^*hypo/hypo*^) at 0, 3 days, 4 weeks, and 8 weeks post-MI. Quantification of the left ventricular (LV) ejection fraction (EF: **A**), fractional shortening (FS: **B**), end-diastolic volume (LVEDV: **C**), end-systolic volume (LVESV: **D**), internal diameter, diastole (LVIDd: **E**), and internal diameter, systole (LVIDs: **F**) is shown. *N* = 10–21 per group. Two-way repeated-measures ANOVA with Bonferroni post hoc test for 2 groups over time. **P < 0.01 or ***P < 0.001 vs. sham for each genotype (denoted by different colors for sham within same group); ^#^*P* < 0.05 or ^##^*P* < 0.01 vs^.^ MI WT (denoted by blue); and ^§§^*P* < 0.01 or ^§§§^*P* < 0.001 vs. MI miR-150 KO (denoted by brown). Data are presented as the mean ± SD.

Data are presented as the mean ± SEM (except Fig. 1 using SD because no clear variation bars are shown otherwise) from independent experiments with different biological samples per group. Triplicate experiments were performed for all biochemical and cell biology studies. The number of in vitro biological samples per group was 3–6. The number of mouse samples per group was 3–18. The exact sample size for each experimental group/condition is given as a number in the figure/table legend. To ensure the robustness of the data and to allow the direct evaluation of the distribution of the data, we present graphical data as scatter/dot plots. Normality was assessed with the Kolmogorov-Smirnov test. The following statistical tests were used: unpaired two-tailed t-test for comparisons between 2 groups, one-way ANOVA with Tukey’s multiple comparison test for multiple groups, two-way ANOVA with Tukey’s multiple comparison test for comparisons between 2 groups with different treatments, and two-way repeated-measures ANOVA with Bonferroni’s post hoc test for 2 groups over time. The unpaired 2-tailed t-test was based on assumed normal distributions. A *P* value < 0.05 was considered statistically significant. *P* values are indicated as follows: *^,#,or§^*P* < 0.05, **^,##,or§§^*P* < 0.01, and ***^,###,or§§§^*P* < 0.001.

## Results

### Sprr1a knockdown in miR-150 KO mice largely corrects cardiac dysfunction mediated by miR-150 deletion

*Sprr1a* is a direct target of miR-150 in vitro, miR-150 acts as a gatekeeper of CM survival in part by inhibiting proapoptotic *Sprr1a* [13], and their correlative cardiac actions are shown [12, 13]; but an in vivo functional relationship between miR-150 and *Sprr1a* in the heart has not been established. To directly investigate their in vivo functional interaction in the heart, we generated a novel miR-150 KO;*Sprr1a*^*hypo/hypo*^ mouse line by breeding miR-150 KO mice with *Sprr1a*^*hypo/hypo*^ mice. We first conducted permanent ligation of the left anterior descending (LAD) artery in mice to induce MI. Consistent with a previous report [12], we observe that miR-150 KO mice exhibit normal cardiac function at baseline (Supplementary Table 1 and Fig. 1) but respond differently to MI. Cardiac function is significantly compromised in miR-150-null mice following MI. First, MI significantly worsens the cardiac function of miR-150 KO mice at 3 days as indicated by a decreased ejection fraction (EF), fractional shortening (FS), diastolic left ventricular anterior wall thickness (LVAW), and systolic left ventricular posterior wall thickness (LVPW) as well as an increase in end-systolic volume (ESV) and systolic left ventricular internal diameter (LVID) compared to those of WT controls (Supplementary Table 2 and Fig. 1). MiR-150 KO mice also display impaired cardiac function at 4 weeks post-MI, shown by a significant decrease in EF, FS, diastolic LVPW, and systolic LVPW as well as a significant increase in end-diastolic volume (EDV), ESV, diastolic LVID, and systolic LVID (Supplementary Table 3 and Fig. 1). MI also causes augmented cardiac dysfunction in miR-150 KO mice at 8 weeks as evidenced by a significant decrease in EF, FS, diastolic LVAW, diastolic LVPW, and systolic LVPW as well as a significant increase in EDV, ESV, diastolic LVID, and systolic LVID (Supplementary Table 4 and Fig. 1). In contrast, WT controls show less functional impairment at 4 weeks (Supplementary Table 3 and Fig. 1) and 8 weeks following MI (Supplementary Table 4 and Fig. 1).

We next show that miR-150 KO;*Sprr1a*^*hypo/hypo*^ mouse hearts are functionally normal at baseline (Supplementary Table 1 and Fig. 1). However, a significant improvement in cardiac function at 3 days after MI is observed in miR-150 KO;*Sprr1a*^*hypo/hypo*^ mice compared to miR-150 KO mice, indicated by an increase in cardiac output (CO), EF, FS, and diastolic LVAW as well as a decrease in EDV, ESV, diastolic LVID, and systolic LVID (Supplementary Table 2 and Fig. 1). MiR-150 KO;*Sprr1a*^*hypo/hypo*^ mice also display enhanced cardiac function at 4 weeks post-MI as evidenced by a significant increase in EF, FS, diastolic LVAW, systolic LVAW, diastolic LVPW, and systolic LVPW as well as a significant decrease in EDV, ESV, diastolic LVID, and systolic LVID (Supplementary Table 3 and Fig. 1) compared to those of miR-150 KO mice. Last, we show improved cardiac function in miR-150 KO;*Sprr1a*^*hypo/hypo*^ mice at 8 weeks post-MI compared to miR-150 KO mice as shown by a significant increase in CO, EF, FS, heart rate (HR), diastolic LVAW, systolic LVAW, and systolic LVPW as well as a significant decrease in EDV, ESV, diastolic LVID, and systolic LVID (Supplementary Table 4 and Fig. 1). Our morphometric data also show that miR-150 KO;*Sprr1a*^*hypo/hypo*^ mice have a significant decrease in the ratio of heart weight/body weight (HW/BW) and the ratio of left ventricle weight/body weight (LVW/BW) at 8 weeks after MI compared to miR-150 KO controls (Supplementary Table 4). Notably, we do not observe any difference in post-MI mortality between groups (Supplementary Tables 1, 3, and 4: see n for animal numbers per each group at week 0, week 4, and week 8 after MI).

### Sustained Sprr1a knockdown alleviates cardiac damage, inflammation, and apoptosis post-MI mediated by miR-150 loss

**Fig. 2:**
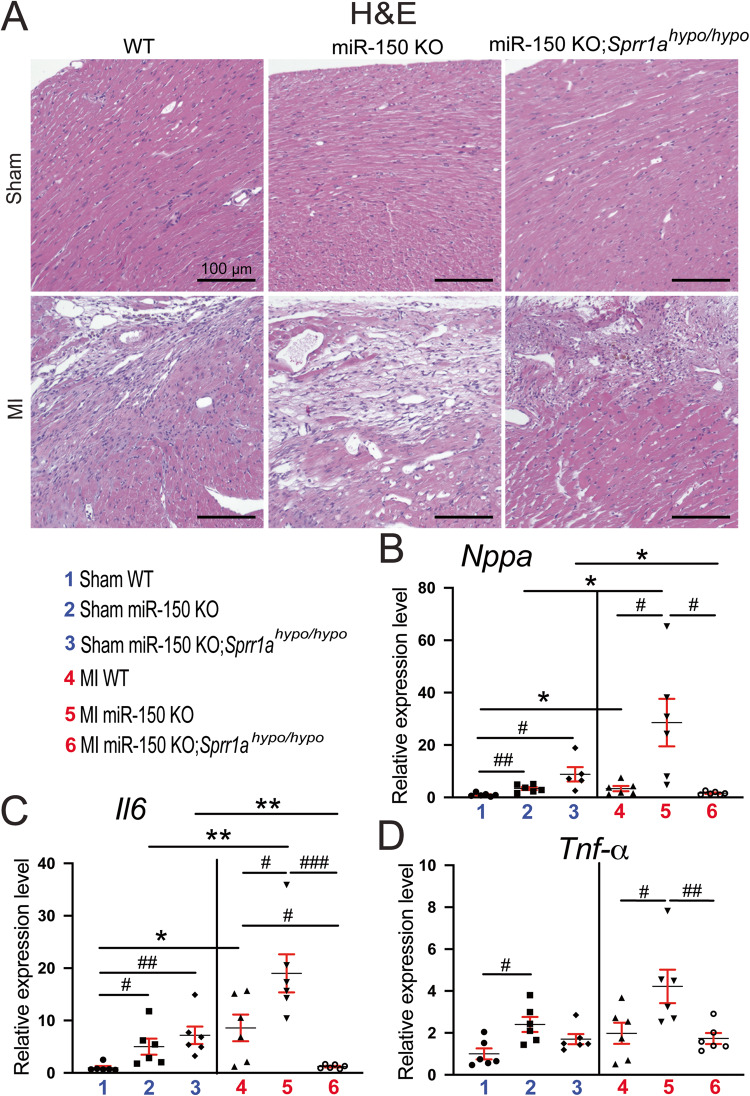
Downregulation of *Sprr1a* suppresses cardiac damage and inflammation post-myocardial infarction in miR-150 KO mice. **A** Representative hematoxylin and eosin (H&E) staining of heart sections of the peri-ischemic border area from the 6 experimental groups at 8 weeks post-MI shows a decrease in the loss of normal architecture and cellular integrity as well as in disorganized structure in miR-150 KO;*Sprr1a*^*hypo/hypo*^ hearts compared to miR-150 KO controls. Scale bars: 100 μm. **B** qRT-PCR analysis of *Nppa* expression representing cardiac damage in ischemic areas from WT, miR-150 KO, and miR-150 KO;*Sprr1a*^*hypo/hypo*^ mouse hearts at 8 weeks post-MI. qRT-PCR analysis of inflammatory *Il6* (**C**) and *Tnf-a* (**D**) expression in ischemic areas from WT, miR-150 KO, and miR-150 KO;*Sprr1a*^*hypo/hypo*^ mouse hearts at 8 weeks post-MI. *N* = 5–6 per group. qRT-PCR data (**B**–**D**) are shown as the fold induction of gene expression normalized to *Gapdh*. Two-way ANOVA with Tukey’s multiple comparison test. *P < 0.05 or **P < 0.01 vs. sham for each genotype; ^#^*P* < 0.05, ^##^*P* < 0.01, or ^###^*P* < 0.001 vs. WT or miR-150 KO. Data are presented as the mean ± SEM.

**Fig. 3:**
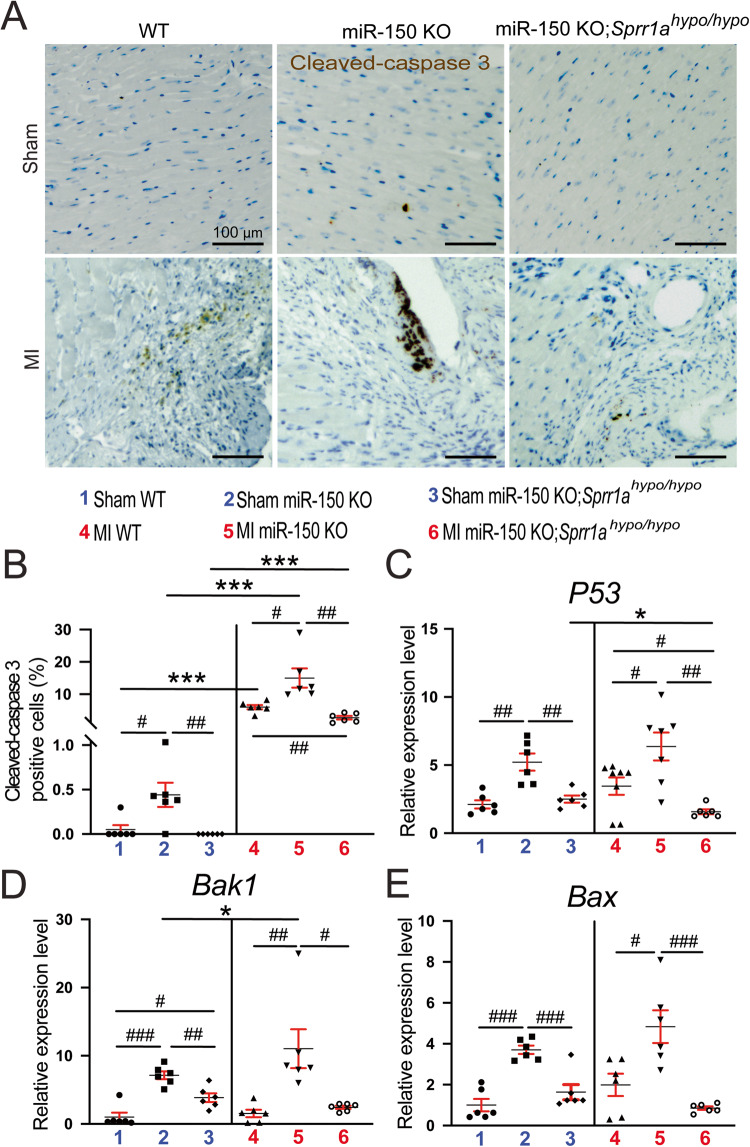
*Sprr1a* knockdown alleviates cardiac apoptosis after myocardial infarction caused by miR-150 deletion. Representative cleaved caspase-3 staining images in heart sections of the peri-ischemic border area in WT, miR-150 KO, and miR-150 KO;*Sprr1a*^*hypo/hypo*^ hearts at 8 weeks post-MI (**A**) and quantification of apoptosis in six 40X fields (**B**). Scale bars: 100 μm. qRT-PCR analysis of proapoptotic *p53* (**C**), *Bak1* (**D**), or *Bax* (**E**) expression in the ischemic areas from WT, miR-150 KO, and miR-150 KO;*Sprr1a*^*hypo/hypo*^ mouse hearts at 8 weeks post-MI. Data are shown as the fold induction of gene expression normalized to *Gapdh*. *N* = 6 per group. Two-way ANOVA with Tukey’s multiple comparison test. *P < 0.05 or ***P < 0.001 vs. sham for each genotype; ^#^*P* < 0.05, ^##^*P* < 0.01, or ^###^*P* < 0.001 vs. WT or miR-150 KO. Data are presented as the mean ± SEM.

We previously reported that miR-150 KO mice display excessive maladaptive post-MI remodeling, such as cardiac damage, inflammation, and apoptosis [12]. To determine whether repression of *Sprr1a* mediates the major functions of miR-150 in vivo, we employed miR-150 KO;*Sprr1a*^*hypo/hypo*^ mice and assessed post-MI remodeling compared to that of miR-150 KO controls. We find that miR-150 KO;*Sprr1a*^*hypo/hypo*^ hearts exhibit a decrease in the loss of normal architecture and cellular integrity (Fig. 2A) as well as decreased mRNA levels of fetal *Nppa* (Fig. 2B) after 8 weeks of MI compared to miR-150 KO hearts. We next examined whether an improved cardiac inflammatory cell (CI) response contributes to the decreased disorganized structure in miR-150 KO;*Sprr1a*^*hypo/hypo*^ hearts post-MI. Notably, inflammatory *Il-6*, *Tnf-α*, and *Ptprc* are also downregulated in miR-150 KO;*Sprr1a*^*hypo/hypo*^ hearts (Fig. 2C, D and Supplementary Fig. 1) compared to miR-150 KO hearts post-MI. Finally, we find that miR-150 KO;*Sprr1a*^*hypo/hypo*^ hearts contain significantly lower numbers of cleaved caspase-3-positive cells (Fig. 3A, B), indicating decreased apoptosis in miR-150 KO;*Sprr1a*^*hypo/hypo*^ hearts. Our data further show that miR-150 KO;*Sprr1a*^*hypo/hypo*^ hearts have decreased mRNA levels of apoptotic *P53*, *Bak1*, and *Bax* (Fig. 3C–E) compared to levels in miR-150 KO hearts. Altogether, our data suggest that sustained *Sprr1a* downregulation ameliorates adverse post-MI remodeling caused by miR-150 deletion and that miR-150 is a functionally important upstream negative regulator of *Sprr1a* in the heart.

### Knockdown of Sprr1a in miR-150 KO mice blunts cardiac fibrosis post-MI observed following miR-150 deficiency

**Fig. 4:**
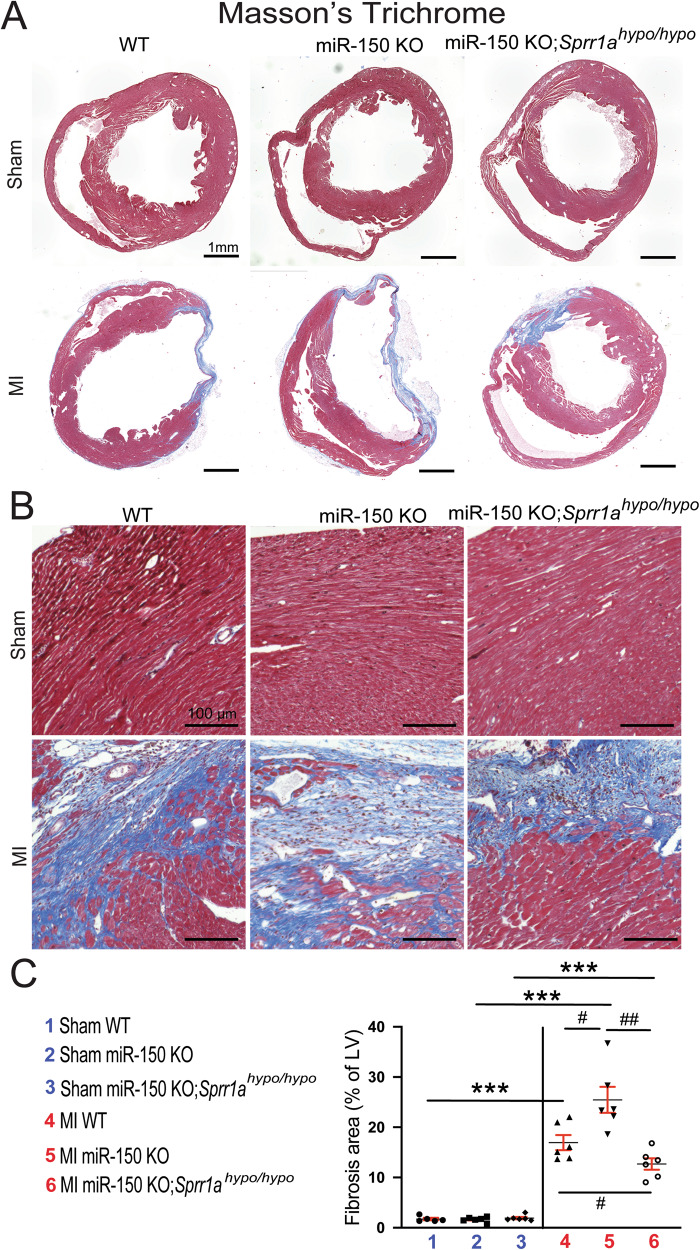
MiR-150 decreases cardiac fibrosis post-myocardial infarction in part by repressing *Sprr1a*. Representative Masson’s trichrome staining (**A**, **B**) in heart sections of the peri-ischemic border area in the 6 experimental groups at 8 weeks post-MI and fibrosis quantification (**C**) in whole left ventricles (LVs). Fibrosis histology images from whole heart longitudinal sections (**A**: Scale bars: 1 mm) and zoomed in images of the peri-ischemic border area (**B**: Scale bars: 100 μm). *N* = 6 per group. Two-way ANOVA with Tukey’s multiple comparison test. ***P < 0.001 vs. sham for each genotype; ^#^*P* < 0.05 or ^##^*P* < 0.01 vs. WT or miR-150 KO. Data are presented as the mean ± SEM.

**Fig. 5:**
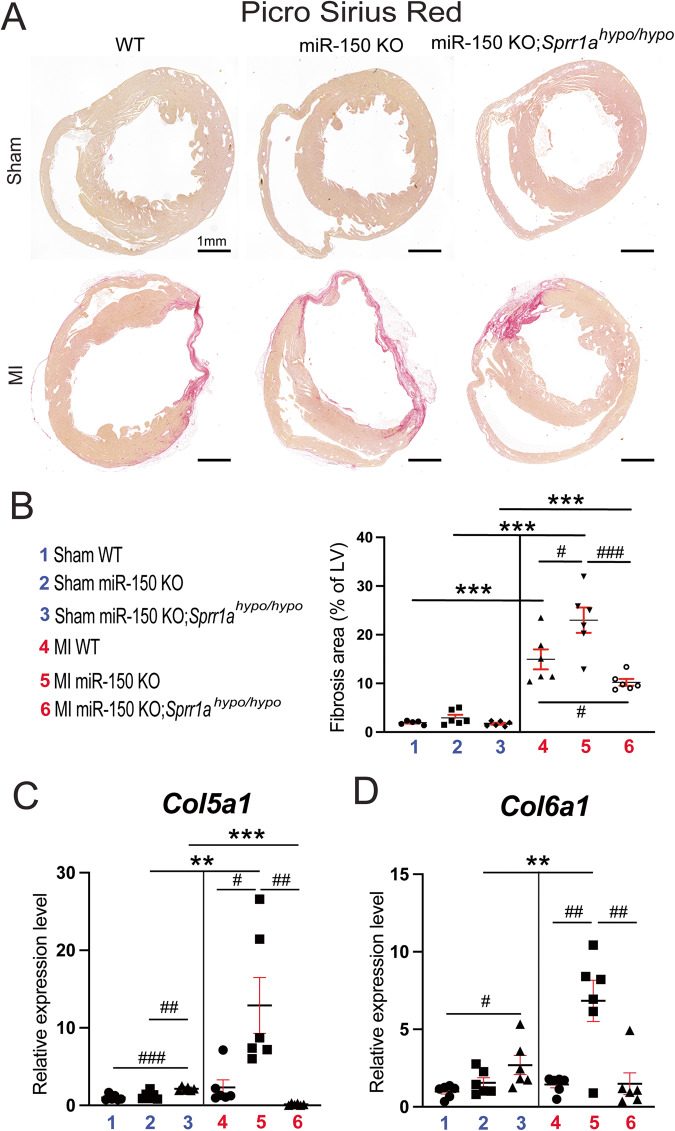
MiR-150 reduces cardiac fibrosis as well as the expression of *Col5a1* and *Col6a1* after myocardial infarction in part by suppressing *Sprr1a*. Representative picrosirius red staining (**A**) from heart sections in the 6 experimental groups at 8 weeks post-MI and fibrosis quantification (**B**) in whole left ventricles (LVs). Fibrosis histology images from whole heart longitudinal sections (**A**: Scale bars: 1 mm) are shown. *N* = 6 per group. Two-way ANOVA with Tukey’s multiple comparison test. ***P < 0.001 vs. sham for each genotype; ^#^*P* < 0.05 or ^###^*P* < 0.001 vs. WT or miR-150 KO. Data are presented as the mean ± SEM. qRT-PCR analysis of profibrotic *Col5a1* (**C**) or *Col6a1* (**D**) expression in ischemic areas from WT, miR-150 KO, and miR-150 KO;*Sprr1a*^*hypo/hypo*^ mouse hearts at 8 weeks post-MI. Data are shown as the fold change of gene expression normalized to *Gapdh*. *N* = 6 per group. Two-way ANOVA with Tukey’s multiple comparison test. **P < 0.01 or ***P < 0.001 vs. sham for each genotype; ^#^*P* < 0.05, ^##^*P* < 0.01, or ^###^*P* < 0.001 vs. WT or miR-150 KO. Data are presented as the mean ± SEM.

**Fig. 6:**
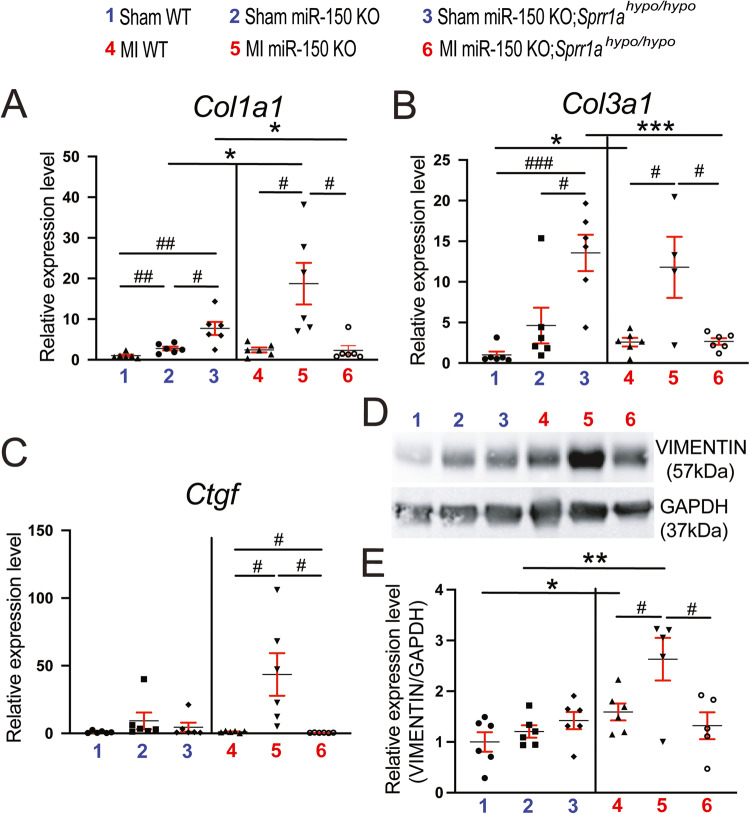
*Sprr1a* is a significant functional target for miR-150 repression in mouse hearts and induces fibrotic markers. qRT-PCR analysis of profibrotic *Col1a1* (**A**), *Col3a1* (**B**), or *Ctgf* (**C**) expression in ischemic areas from WT, miR-150 KO, and miR-150 KO;*Sprr1a*^*hypo/hypo*^ mouse hearts at 8 weeks post-MI. Data are shown as the fold induction of gene expression normalized to *Gapdh*. *N* = 4–6 per group. Two-way ANOVA with Tukey’s multiple comparison test. *P < 0.05 or ***P < 0.001 vs. sham for each genotype; ^#^*P* < 0.05, ^##^*P* < 0.01, or ^###^*P* < 0.001 vs. WT or miR-150 KO. Data are presented as the mean ± SEM. **D**, **E** VIMENTIN protein levels were measured in ischemic areas from WT, miR-150 KO, and miR-150 KO;*Sprr1a*^*hypo/hypo*^ mouse hearts at 8 weeks post-MI. *N* = 5–6 per group. Two-way ANOVA with Tukey’s multiple comparison test. *P < 0.05 or **P < 0.01 vs. sham for each genotype; ^#^*P* < 0.05 vs. WT or miR-150 KO. Data are presented as the mean ± SEM.

To further determine the response of miR-150 KO;*Sprr1a*^*hypo/hypo*^ mice to MI, we assessed the degree of fibrosis using Masson’s trichrome staining and picrosirius red staining of the hearts at 8 weeks post-MI. We find larger regions of fibrosis in miR-150 KO hearts than in WT MI controls, as reported previously [12]. We next observe reduced fibrosis post-MI in miR-150 KO;*Sprr1a*^*hypo/hypo*^ hearts compared to miR-150 KO hearts (Figs. 4, 5A, B, and Supplementary Fig. 2). MiR-150 KO MI hearts also exhibit increased expression of fibrotic *Col5a1*, *Col6a1*, *Col1a1*, *Col3a1*, and *Ctgf* (Figs. 5C, D, and 6A–C) compared to expression in WT controls, but miR-150 KO;*Sprr1a*^*hypo/hypo*^ MI hearts exhibit decreased expression of these profibrotic genes (Figs. 5C, D, and 6A–C) compared to miR-150 KO controls. Next, our in vivo protein analysis reveals significantly elevated levels of VIMENTIN and α-SMA in miR-150 KO MI mouse hearts compared to WT controls and significantly decreased levels of VIMENTIN and α-SMA in miR-150 KO;*Sprr1a*^*hypo/hypo*^ hearts at 8 weeks post-MI compared to miR-150 KO controls (Fig. 6D, E, and Supplementary Fig. 3); this is consistent with the mRNA data for the profibrotic genes (Figs. 5C, D, and 6A–C). Collectively, these results demonstrate for the first time that genetic knockdown of *Sprr1a* significantly attenuates adverse postinfarct remodeling mediated by miR-150 deletion.

### MiR-150 in human CFs elicits protective effects in part through direct functional repression of profibrotic SPRR1A

Because of the cardiac upregulation of miR-150 by Carv [11] concurrent with the downregulation of *Sprr1a* [13], and the downregulation of miR-150 in CFs isolated from TAC mice [15] concurrent with the upregulation of *Sprr1a* in CFs during MI [13], we next studied primary adult human CFs (HCFs) to test whether miR-150 and *SPRR1A* are inversely regulated in HCFs treated with Carv as well as HCFs subjected to H/R conditions. Indeed, *SPRR1A* is downregulated in HCFs subjected to H/R conditions after Carv treatment (Supplementary Fig. 4) concurrent with the upregulation of miR-150 [28]. We also observe that *SPRR1A* is increased in HCFs after H/R (Supplementary Fig. 4), consistent with our in vivo results in post-MI hearts and isolated CFs from ischemic myocardium [13]. Notably, we previously reported that miR-150 is downregulated in HCFs after H/R [28]. Together with other previous reports on miR-150 downregulation in H/R and MI [12] as well as I/R [29, 30], our results indicate that *Sprr1a* is a critical functional target of miR-150 in CFs.

**Fig. 7:**
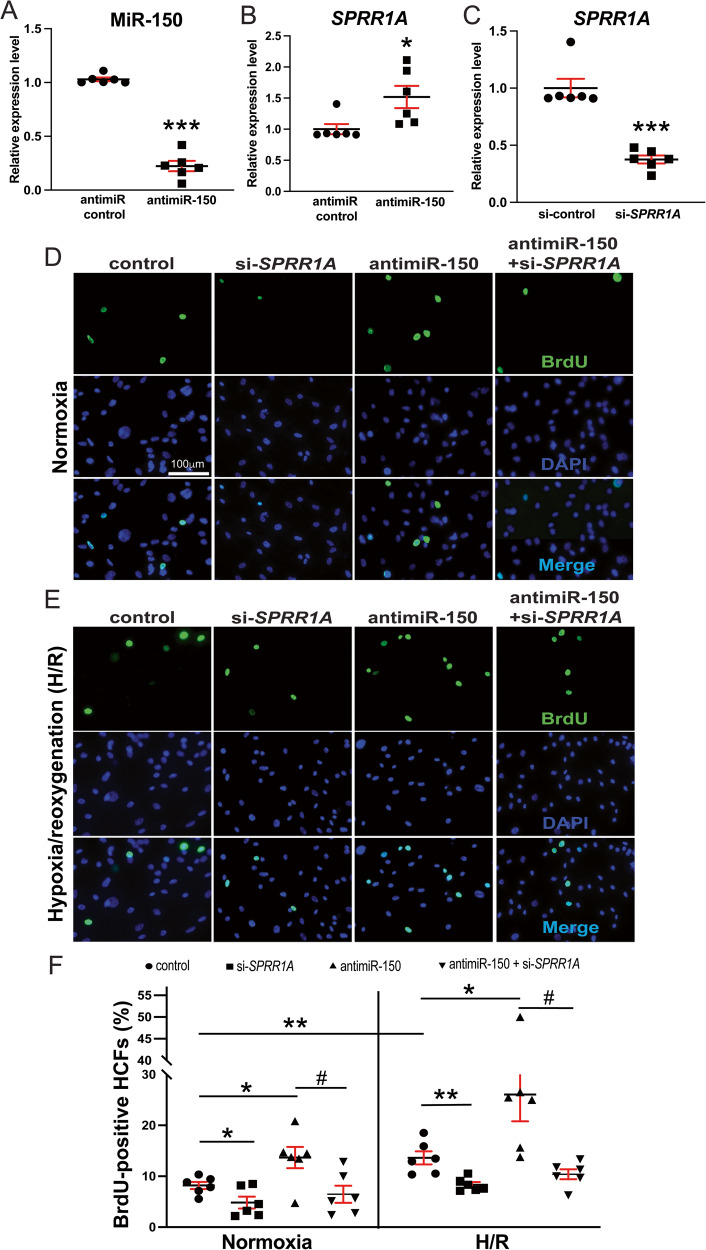
*SPRR1A* is repressed by miR-150 in human cardiac fibroblasts (HCFs) and is necessary for miR-150-dependent regulation of HCF proliferation. HCFs were transfected with antimiR control or antimiR-150 (**A**, **B**) and with control scramble siRNA (si-control) or *SPRR1A* siRNA (si-*SPRR1A*) (**C**). qRT-PCR analyses for miR-150 (**A**) or *SPRR1A* (**B**, **C**) were then performed to check their expression after the indicated transfection. Data were normalized to *U6 SNRNA* (**A**) or *GAPDH* (**B**, **C**) and are expressed relative to controls. *N* = 6 per group. Unpaired 2-tailed t-test. RNA interference with *SPRR1A* protects HCFs from the increased proliferation mediated by antimiR-150. HCFs were transfected as indicated and subjected to normoxia or hypoxia/reoxygenation (H/R). Bromodeoxyuridine (BrdU) assays were then performed under both normoxic (**D**, **F**) and H/R (**E**, **F**) conditions. The percentage of proliferating nuclei (green) was calculated by normalizing to the total nuclei (blue). *N* = 6 per group. One-way ANOVA with Tukey’s multiple comparison test. *P < 0.05 or **P < 0.01 vs. control: either si-control or antimiR control. ^#^*P* < 0.05 vs. anti-miR-150. Data are presented as the mean ± SEM.

Because *Sprr1a* expression is upregulated in CFs isolated from ischemic mouse hearts [13] concurrent with the downregulation of miR-150 in CFs isolated from TAC mice [15], and miR-150 negatively regulates mouse CF activation in vitro [15], we first confirmed whether a direct target of miR-150, *SPRR1A* is repressed by miR-150 in HCFs. Our loss-of-function studies indeed show that *SPRR1A* is increased after miR-150 inhibition in HCFs (Fig. 7A, B). We next investigated whether *SPRR1A* regulates HCF activation. We first observe that *SPRR1A* knockdown in HCFs decreases the expression of profibrotic *ACTA2* and *CTGF* (Fig. 7C and Supplementary Fig. 5), and miR-150 knockdown increases the expression of *ACTA2, CTGF*, and *POSTN* (Supplementary Fig. 6).

**Fig. 8:**
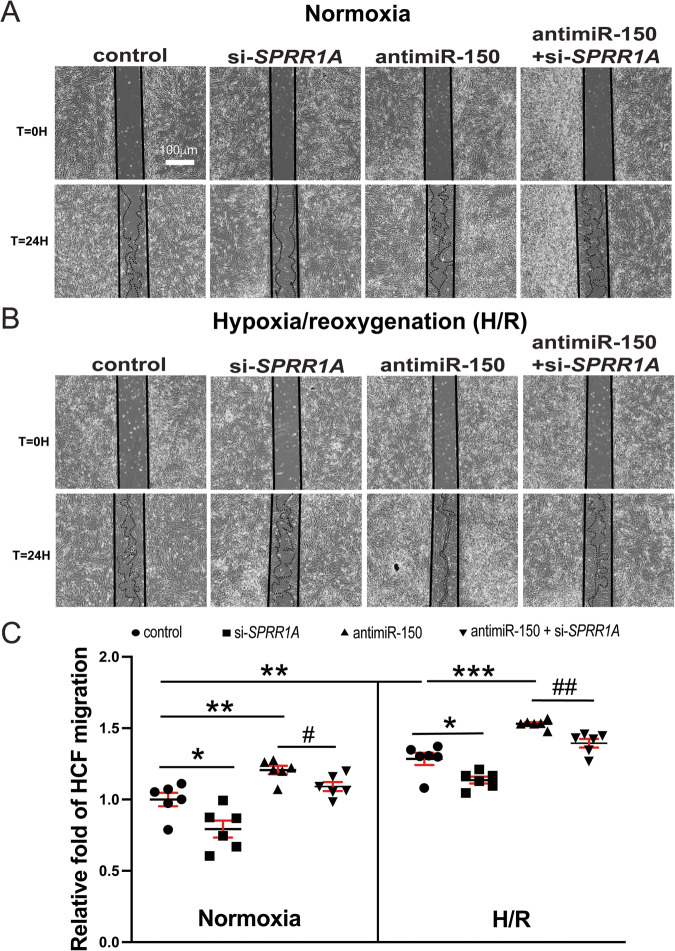
*SPRR1A* is necessary for miR-150-dependent regulation of HCF migration. **A–C** HCFs were transfected and subjected to normoxia or hypoxia/reoxygenation (H/R) as indicated in Fig. 7D–F. Scratch migration assays were then performed. RNA interference with *SPRR1A* protects HCFs from the increased migration mediated by antimiR-150. *N* = 6 per group. Two-way repeated-measures ANOVA with Bonferroni post hoc test. One-way ANOVA with Tukey’s multiple comparison test. *P < 0.05, **P < 0.01, or ***P < 0.001 vs. control: either si-control or anti-miR control. ^#^*P* < 0.05 or ^##^*P* < 0.01 vs. anti-miR^-^150. Data are presented as the mean ± SEM.

To further assess the effects of *SPRR1A* knockdown, we examined HCF proliferation using bromodeoxyuridine assay. We find that compared to controls, *SPRR1A* knockdown decreased HCF proliferation (Fig. 7D–F) under both normoxic and H/R conditions. This is consistent with our gene expression data, showing that HCFs with *SPRR1A* knockdown have decreased mRNA levels of S-phase marker *PCNA*, mitosis (M) marker *AURKB*, and G2/M-phase marker CCNB1 compared with controls (Supplementary Fig. 7). Moreover, our wound migration studies reveal that compared to controls, *SPRR1A* knockdown decreased HCF migration (Fig. 8A–C) under both normoxic and H/R conditions. This is consistent with our gene expression data, showing that *SPRR1A* knockdown in HCFs subjected to H/R decreases mRNA levels of cell migration markers, *CTHRC1* and *TNC* compared with controls (Supplementary Fig. 8). *SPRR1A* knockdown in HCFs also suppresses mRNA levels of CF differentiation markers, *COL4A1*, *COL8A1*, and *SRF* (Supplementary Fig. 9), as well as the protein levels of profibrotic α-SMA and FIBRONECTION (Supplementary Fig. 10). Because TGF-β1/SMAD signaling pathway plays a key role in CF activation, we next investigated the role of SPRR1A in the regulation of TGF-β1 and SMADs. We observe that *SPRR1A* knockdown in HCFs subjected to H/R decreases mRNA levels of *TGFB1*, *SMAD2*, and *SMAD3* compared with controls (Supplementary Fig. 11). This is consistent with our in vivo data, showing that *Sprr1a* knockdown in mice decreases *Smad3* expression as well as mRNA and protein levels of TGF-β1 compared with controls (Supplementary Figs. 12, 13). Our data thus suggest that SPRR1A is sufficient to increase HCF activation in part by activating TGF-β1/SMAD signaling pathway.

Finally, to establish the functional relationship between miR-150 and *SPRR1A* in HCF activation, we applied an antimiR/siRNA-based rescue strategy to validate the functional relevance of the direct miR-150 target *SPRR1A*. MiR-150 knockdown increases HCF proliferation (Fig. 7D–F and Supplementary Fig. 7) and migration (Fig. 8A–C and Supplementary Fig. 8), which are attenuated by siRNA against *SPRR1A* (Figs. 7D–F, 8A–C, Supplementary Figs. 7, 8). We also show that miR-150 knockdown increases the expression of profibrotic *TGFB1*, *SMAD2, SMAD3, COL1A1, COL3A1*, *COL4A1*, *COL8A1*, and *SRF* under normoxic and/or H/R conditions, which are attenuated by *SPRR1A* knockdown (Supplementary Figs. 9, 11, 14). Taken together, our data indicate that profibrotic SPRR1A is a key direct and functional target of miR-150 in HCFs and whole mouse hearts.

## Discussion

In this study, we identify the direct functional interaction between miR-150 and SPRR1A as a new regulatory mechanism pertinent to both MI in mice and HCF activation. Mice deficient in miR-150 are sensitized to MI as indicated by the increased cardiac fibrosis, apoptosis, inflammation, and damage as well as impairment of left ventricular function. Using a novel miR-150 KO;*Sprr1a*^*hypo/hypo*^ mouse model, we demonstrate that *Sprr1a* knockdown attenuates excessive adverse postinfarct remodeling mediated by miR-150 loss. Of note, miR-150 KO;*Sprr1a*^*hypo/hypo*^ rescues in part the phenotype exhibited in miR-150 KO, which reaches similar levels shown in WT MI or higher levels than WT MI (Figs. 2–6 and Supplementary Figs. 1, 3). Using primary adult HCFs, we also discover for the first time that miR-150 functionally inhibits profibrotic *SPRR1A* such that the increased expression of *SPRR1A* in HCFs lacking miR-150 results in a higher degree of sustained CF activation. Thus, the current study suggests that SPRR1A is a crucial ischemic injury-responsive target of miR-150 in whole mouse hearts and HCFs and that miR-150 confers protective actions on both HF in mice and HCF activation by repressing profibrotic SPRR1A.

β_1_AR is predominantly expressed in the heart, and β-arrestin-mediated β_1_AR signaling elicits protective effects after Isoproterenol (ISO)-induced injury [31]. In our previous study, miR-150 was activated by the β-blocker Carv acting through β-arrestin-mediated β_1_AR protective signaling [11]. We recently reported that miR-150 is a critical downstream mechanism by which β_1_AR-mediated β-arrestin signaling pathways confer protection [32]. Together with the results presented here, we posit that β-arrestin-mediated β_1_AR regulatory mechanisms of miR-150 activation elicit beneficial remodeling in failing hearts by repressing CF activation through inhibiting profibrotic genes, including *Sprr1a*. Interestingly, we previously showed that miR-150 deletion significantly compromised cardiac function and remodeling following MI by increasing cell death without affecting neovascularization [12], whereas Liu Z et al. reported that miR-150 overexpression mediated by AgomiR injection protected the mouse heart against acute MI (AMI) by inhibiting monocyte migration [20]. More recently, using a novel mouse model, we demonstrated that cardiac-specific miR-150 cKO mice had enhanced maladaptive post-MI remodeling. Mechanistically, miR-150 represses a proapoptotic and direct CM target, *Sprr1a* [13]. We also showed that *Sprr1a*^*hypo/hypo*^ mice are protected against MI [13]. Although these previous studies showed the correlative relationship between miR-150 and *Sprr1a* in HF, our overall knowledge of their functional actions remains elusive in part because of (i) the lack of mechanistic insight by which the miR-150/*Sprr1a* dyad regulates HF and (ii) the absence of rigorous studies to establish their direct in vivo functional relationship in HF. Our previous fractionation studies of different cardiac cell types showed that the expression of miR-150 was significantly higher in normal CMs than CFs at baseline and its expression was not altered in stressed CMs, CFs, CIs, and cardiac endothelial cells (CEs) isolated from ischemic myocardium at 1 week post-MI [13]. Other studies reported that cardiac-specific overexpression of miR-150 alleviates TAC-induced cardiac hypertrophy and HF [14] and that miR-150 loss results in a higher degree of cardiac fibrosis after TAC and miR-150 is downregulated in CFs (not CMs) isolated from TAC mice [15]. MiR-150 also negatively regulates mouse CF activation in vitro [15]. It is known that TGF-β1 or TGF-β/Smad signaling inhibits miR-150 expression [33, 34], coincident with SPRR1A upregulation [35]. MiR-150 can also suppress TGF-β in rat hearts and primary CFs [36]. These previous studies indicate that miR-150 or SPRR1A plays an antifibrotic or profibrotic role, respectively. Interestingly, prior studies showed that SRF and c-Myb are possible downstream mechanisms that explain antifibrotic effects of miR-150 in the heart [14, 15]. Liu W et al. reported that SRF is repressed by miR-150 in mouse hearts without presenting any functional rescue experiments [14]. Deng P et al. showed that c-Myb is a key functional target of miR-150 in mouse CFs, but they did not establish their in vivo functional link [15]. Thus, the significance of SRF- and c-Myb-dependent mechanisms of miR-150 actions in vivo has not been defined. Here, we make novel discoveries to establish the functional miR-150/SPRR1A axis in whole mouse hearts and HCFs as well as to define the role of SPRR1A in CF activation.

SPRR1A is a known substrate of TGase II, and prior studies have linked TGase II to HF [37, 38] and apoptosis of noncardiac cells [39, 40], suggesting a potential role of SPRR1A in HF and apoptosis. In agreement with this idea, we previously showed that CMs with knockdown of *Sprr1a* are protected against apoptosis in H/R and that *Sprr1a*^*hypo/hypo*^ mice are protected against MI [13]. Interestingly, *Sprr1a* expression is increased in post-MI hearts [13, 23] concurrent with miR-150 downregulation [12]. Our previous cardiac cell fractionation study showed that *Sprr1a* was ubiquitously expressed in CMs, CFs, CIs, and CEs at baseline. Despite this ubiquitous expression pattern at baseline, *Sprr1a* is upregulated in CMs isolated from mouse hearts post-MI [13], consistent with a report of SPRR1A upregulation in CMs from TAC-induced myocardium [23]. Of importance, we also showed that *Sprr1a* is upregulated in CFs isolated from post-MI hearts [13], suggesting a potential role of *Sprr1a* in CFs and cardiac fibrosis. This notion is supported by previous studies showing that adenovirus-mediated ectopic overexpression of *Sprr1a* in vivo promotes cardiac fibrosis after TAC [23] and that SPRR1A levels are more highly expressed in CFs from the infarct zone than in those from the remote region following MI [27]. Our current study also demonstrates for the first time that *SPRR1A* knockdown suppresses HCF proliferation and migration (Figs. 7, 8 and Supplementary Figs. 7, 8) and that *Sprr1a* knockdown attenuates cardiac fibrosis post-MI observed following miR-150 deletion (Figs. 4–6 and Supplementary Figs. 2,3). In our previous study, we also reported that LV *SPRR1A* is upregulated in patients with HFrEF [13], consistent with other studies in mice with ISO-induced myocardial injury [24] and renal I/R injury [25]. Notably, *SPRR1A* expression was inversely associated with the survival of cancer patients [41–43]. Moreover, our recent study reported that *Sprr1a* is downregulated in hearts and CMs by Carv [13], concurrent with the upregulation of miR-150 [11]. Here, we also show that *SPRR1A* is downregulated in HCFs by Carv (Supplementary Fig. 4), concurrent with the upregulation of miR-150 [28]. Consistent with our findings, the cardiac upregulation of *Sprr1a* after injury is suppressed by treatment with cardioprotective Danshen [24]. Thus, the findings of *Sprr1a* upregulation in CMs and CFs during MI further support that *Sprr1a* inhibition could be therapeutically beneficial for HF and cardiac fibrosis. Notably, a regeneration-associated gene, *Sprr1a* was shown to be regulated by miR-463-3p in tibial nerve tissue [44] as well as by miR-155 in neurons and macrophages [45]. Except for these two other reports, little is known about the regulation of *Sprr1a* by miRs. Given our findings that profibrotic SPRR1A is an important functional target of miR-150 in whole mouse hearts (Figs. 1–6 and Supplementary Figs. 1–3) and HCFs (Figs. 7, 8, Supplementary Figs. 7–9, and Supplementary Figs. 11, 14) and that SPRR1A was regulated by miR-150 by direct interaction [13], future targeted treatment options based on SPRR1A could be considered in MI patients with decreased levels of miR-150.

### Limitations

Although we demonstrate that systemic knockdown of *Sprr1a* in mice alleviates maladaptive post-MI remodeling caused by miR-150 loss and that miR-150 is an important negative regulator of HCF activation in vitro by functionally repressing profibrotic *SPRR1A*, miR-150 or *Sprr1a* expression in other myocardial cells may also play a prominent role as supported by our recent study using CM-specific miR-150 cKO mice [13]. Future studies using conditional cell-specific mouse models are thus warranted to fully understand the possible contribution of miR-150 or *Sprr1a* expression in other cell types to postischemic heart remodeling. Especially, whether *Sprr1a* is a key downstream effector of fibroblast miR-150 during post-MI fibrotic remodeling in mice remains to be determined and is beyond the scope of the current study. To expand our understanding of the sequence of events and to evaluate fibroblast roles in inflammatory and wound healing responses, additional immunohistochemical assessments and gene expression studies (e.g., inflammation, acute cardiac cell death, and wound healing) during AMI are also needed. Notably, our functional rescue data in mouse hearts and HCFs establish a functional link between miR-150 and SPRR1A in HF, supporting that they are in a linear pathway. Nevertheless, miR-150 may also have other targets mediating distinct functions. We will investigate additional novel functional targets (i.e., SPRR1A-independent mechanisms) in the heart by cross-referencing the gene signature from cardiac-specific miR-150 cKO mice [13] with miR-150 target prediction analyses in our future mechanistic studies. Importantly, the downstream targets and mechanisms of SPRR1A that regulate CF activation remain elusive despite the suggestion in our current data (Supplementary Figs. 5 and 7–14) that SPRR1A activates profibrotic markers. Especially, a potential role of SPRR1A as a regulator of the cytoskeleton remains to be determined because SPRR1A is localized selectively to actin-rich membrane ruffles and is colocalized with F-actin microfilaments [46]. Moreover, additional methods, such as the Simpson method with multiple B-mode images and pressure-volume loop analysis would be required to measure cardiac function more accurately and to assess diastolic dysfunction. Atomic force microscopy will be also needed to determine the stiffness of ventricular scar tissue post-MI. In the current study, we use a primary adult HCF model to bolster the translational significance. Although this human cell model is an appropriate human cell source for HF remodeling in vitro, the mode of action in mice could be different in human cells. We would employ CFs isolated from the mice used in this study to confirm the miR-150/SPRR1A axis in CFs. Although we show that modulating the miR-150/SPRR1A axis affects HCF activation after H/R, most CFs are activated by recruited inflammatory cells after MI, not by ischemia. We would thus use TGF-β in future CF studies. Last, other in vivo injury models as well as detailed studies on the other roles of the miR-150/SPRR1A dyad in all cell types are warranted before pursuing this axis as a vital therapeutic modality.

## Conclusions

Our results using a novel double loss-of-function mouse model and primary adult HCFs suggest that SPRR1A knockdown attenuates adverse post-MI remodeling mediated by miR-150 deletion and that miR-150 plays a vital protective role in part by blunting CF activation through its direct functional repression of profibrotic SPRR1A. Although miR-150 is associated with HF in humans [21] and the correlative relationship between miR-150 and SPRR1A in the heart has been shown [13], our studies directly establish the functional relationship between miR-150 and SPRR1A during both post-MI remodeling in mice and HCF activation as well as define an underlying mechanism by which miR-150 affects CF activation. Given that upregulation of SPRR1A [13, 23] or downregulation of miR-150 [16, 29, 30, 47] also underlies other forms of cardiac disease, the deleterious action of SPRR1A and the protective action of miR-150 in whole mouse hearts and HCFs are likely applicable to multiple stress settings. Therefore, reducing SPRR1A levels via SPRR1A knockdown and boosting miR-150 levels via Carv or miR-150 overexpression, in part to attenuate CF activation, could be an attractive adjunctive strategy to provide therapeutic benefits.

## Availability of data and materials

All data are included in the manuscript and Supplementary Information. The analytical methods and study materials will be made available to other researchers for the purposes of reproducing the results or replicating the procedures. Other methods are provided in Supplementary Information.

## Supplementary information

Supplementary texts, tables and figures

Reproducibility checklist

Full and uncropped western blots
