# Supplementary material for: SPRR1A is a key downstream effector of MiR-150 during both maladaptive cardiac remodeling in mice and human cardiac fibroblast activation
Source: Cell Death Dis. 2023 Jul 19;14(7):446. doi: 10.1038/s41419-023-05982-y (PMC10356860; doi:10.1038/s41419-023-05982-y)
Supplement: Supplementary file 1 — Supplementary texts, tables and figures [file 41419_2023_5982_MOESM1_ESM.pdf]

Supplementary Information

Supplementary Materials and Methods

Supplementary References

Supplementary Tables 1–4

Supplementary Figures 1–14 and Legends

Online Supporting Data (unedited original blot images)

Supplementary Materials and Methods

Mouse model of myocardial infarction

Eight–16 week-old miR-150 KO, *Sprr1a*$^{hypo/hypo}$, miR-150 KO;*Sprr1a*$^{hypo/hypo}$, and WT mice were subjected to MI as we previously published [1, 2]. Briefly, mice were anesthetized using 1–4% inhaled isoflurane and placed on a heating pad. Mice were intubated and ventilated with oxygen using a PhysioSuite MouseVent™ ventilator (Kent Scientific). The LAD was visualized under a stereoscope and ligated by using an 8-0 nylon suture. Regional ischemia was confirmed by visual inspection of the discoloration of the occluded distal myocardium. Sham-operated animals underwent the same procedure without LAD occlusion. One dose of extended-release buprenorphine (3.25 mg/kg Ethiqa XR; MWI Animal Health) was given subcutaneously immediately before the surgery to provide up to 72 hours of analgesia. We used responses to toe/skin pinch and heart rate for optimal anesthesia and appropriate postoperative monitoring plans.

Transthoracic high-resolution echocardiography

Left ventricular performance was assessed by two-dimensional echocardiography using a Vevo 2100 Ultrasound (Visual Sonics) at baseline (presurgery) and post-MI (3 days, 4 weeks, and 8 weeks) as previously published [1, 2]. We used M-mode tracings to measure the anterior and posterior wall thicknesses at end-diastole and end-systole. The following parameters were also obtained: left ventricular internal diameter (LVID) in either diastole (LVIDd) or systole (LVIDs), end-diastolic volume (EDV), and end-systolic volume (ESV). A single operator blinded to mouse genotypes conducted echocardiography and data analysis. Fractional shortening (FS) was calculated according to the formula FS (%) = ([LVIDd-LVIDs]/LVIDd) X 100. The ejection fraction

(EF) was calculated by EF (%) = ([EDV-ESV]/EDV) X 100. All other LV performance parameters were also obtained as shown in **Supplementary Tables 1–4**.

Histopathological and immunohistochemical analyses

Morphometric analyses of heart sizes and weights were conducted as we previously published [1-3]. Histopathological analyses of heart tissues, such as fibrosis via Masson's trichrome and picrosirius red staining, were performed as described [4, 5]. For gross histological assessment, heart sections were stained with hematoxylin and eosin (H&E). Cardiac sections were also stained for cleaved caspase-3 using the Dako Autostainer Link 48 to assess apoptosis as we previously published [6]. Briefly, we deparaffinized, rehydrated, and subjected cardiac sections to 5 minutes of EDTA antigen retrieval in a pressure cooker, 15 minutes of endogenous enzyme blocking, 60 minutes of primary antibody incubation (cleaved caspase-3, rabbit polyclonal [1:200 dilution], 9661, Cell Signaling), and 30 minutes of Dako EnVision-HRP reagent incubation. We then detected signals by adding the substrate hydrogen peroxide using diaminobenzidine (DAB) as a chromogen, followed by hematoxylin counterstaining. We quantified brown cells as the number of positive cells x 100/total cell infiltrates in 6 random microscopic (20X) fields in each slice.

Primary adult human cardiac fibroblast culture and transfection

We purchased primary adult HCFs from PromoCell (C12375) and maintained them according to the company's recommendation. The primary HCFs, which were isolated from the ventricles of the adult heart, display normal fibroblastic characteristics and cellular markers (i.e., CD90-positive, α-smooth muscle actin-negative, and slow muscle myosin-negative). We used multiple batches of HCFs with passage numbers of 3–5 to further ensure the specificity of the cells used and the normal growth pattern. We cultured the adult primary HCFs in PromoCell Fibroblast Growth Medium 3 (C-23025), which contains basic fibroblast growth factor (bFGF: 1

ng/ml), recombinant human insulin (5 ug/ml), and 10% fetal calf serum (FCS: 0.1 ml/ml) in the supplement. These HCFs were authenticated by the supplier, and they have been monitored regularly for their authenticity and to be negative for mycoplasma contamination. We transfected HCFs with an Accell Non-targeting Control siRNA Pool (Horizon Discovery Ltd, D-001910-10-05) or an Accell SPRR1A siRNA-SMARTpool targeting human *SPRR1A* (Horizon Discovery Ltd, E-020095-00-0005) by using Lipofectamine™ 3000 reagent (Invitrogen) as previously described [3, 7]. To inhibit miR-150 expression in HCFs, we also transfected Ambion AntimiR[TM] microRNA inhibitors (Life Technologies) specific to miR-150 (MH10070) and a miR inhibitor negative control (4464076) using Lipofectamine™ 3000 reagent (Invitrogen) as described previously [3]. We then processed transfected HCFs for quantitative real-time RT-PCR, Bromodeoxyuridine (BrdU) proliferation, and wound migration assays as mentioned below. For Carv stimulation experiments, we stimulated HCFs with Carv (1 µM; Sigma-Aldrich) in serum-free media for 16 hours as described previously [8] and then processed them for hypoxia/reoxygenation as described below.

An in vitro model of hypoxia/reoxygenation (H/R: simulated ischemia reperfusion [sI/R])

We incubated HCFs transfected with miR inhibitors or siRNAs or treated with Carv in an ischemia buffer that contained 118 mM NaCl, 24 mM NaH_2CO_3, 1 mM $NaHPO_4$, 2.5 mM $CaCl_2$, 1.2 mM $MgCl_2$, 20 mM sodium lactate, 16 mM KCl, and 10 mM 2-deoxyglucose (pH 6.2). We then placed HCFs in the hypoxic chamber (5% CO_2, 0.1% O_2) for 3 hours, followed by 18 hours of reperfusion-mimicking conditions (i.e., replacing the ischemic buffer with normal cell medium under normoxic conditions) as previously described [3, 9]. Our previous study also validated this in vitro model, and we showed that the model caused significantly increased apoptosis in primary CFs [10]. Based on our initial optimization experiments in HCFs at multiple time points (2–3 hours of hypoxia and 4–18 hours of reoxygenation), we chose 3 hours of hypoxia and 18 hours of reoxygenation, during which maximum increases in the expression of profibrotic *POSTN* and *COL3A1* were achieved compared to normoxia. We then processed HCFs for

quantitative real-time RT-PCR, western blotting, BrdU staining, and wound migration assays.

Quantitative real-time RT-PCR

We prepared total RNAs from HCFs and the infarct area of mouse hearts using TRIzol Reagent (Invitrogen) and treated the samples with RNase-free DNase I as previously described [11, 12]. To measure mature miR-150, we used the TaqMan MicroRNA Reverse Transcription Kit (Life Technologies) to generate cDNAs and the miR-150 TaqMan probe (000473; Life Technologies) to measure the evolutionarily conserved mature miR-150 by real-time RT-PCR. We used the U6 snRNA probe 001973 (Life Technologies) as an endogenous control. We generated cDNAs for genes using SuperScript IV reverse transcriptase (Invitrogen) and random hexamers. We detected the expression of genes using TaqMan expression assays for mice (*Nppa*, Mm01255747_g1; *Ctgf*, Mm01192933_g1; *Col1a1,* Mm00801666_g1; *Col3a1,* Mm00802300_m1; *Col5a1,* Mm00489299_m1; *Col6a1,* Mm00487160_m1; *Tgfb1,* Mm01178820_m1; *Smad3*, Mm01170760_m1; *Tnf-α*, Mm00443258_m1; *Il6*, Mm00446190_m1; *Ptprc*, Mm01293577_m1; *p53*, Mm01731290_g1; *Bak1*, Mm00432045_m1; *Bax*, Mm00432051_m1; and *Gapdh*, Mm99999915_g1 for an endogenous control) and humans (*SPRR1A*, Hs00954595_s1, *ACTA2*, Hs00426835_g1; *POSTN,* Hs01566750_m1; *COL1A1*, Hs00164004_m1; *COL3A1*, Hs00943809_m1; *COL4A1*, Hs00266237_m1; *COL8A1*, Hs00156669_m1; *SRF*, Hs01065256_m1; *CTGF,* Hs00170014_m1; *TGFB1,* Hs00998133_m1; *SMAD2,* Hs00998187_m1; *SMAD3*, Hs00969210_m1; *PCNA*, Hs00427214_g1; *AURKB*, Hs00945858_g1; *CCNB1,* Hs01030099_m1; *CTHRC1*, Hs00298917_m1; *TNC*, Hs01115665_m1; and *GAPDH*, Hs02786624_g1 as an endogenous control). We analyzed quantitative real-time PCRs using a QuantStudio 3 Detection System (Life Technologies) as described previously [12]. We calculated the expression compared to endogenous controls using $2^{-\Delta\Delta Ct}$ and normalized the expression levels to the control.

Western blotting and antibodies

We washed the left ventricles, including the ischemic and border areas and HCFs once with PBS and solubilized them in Nonidet P-40 lysis buffer as previously described [1]. We resolved lysate samples by SDS-PAGE and transferred them to PVDF membranes (Bio-Rad) for immunoblotting. We purchased and used α-SMA (ab7817, mouse, Abcam), VIMENTIN (sc-6260, mouse, Santa Cruz), FIBRONECTIN (sc-8422, mouse, Santa Cruz), SPRR1A (ab125374, rabbit, Abcam), TGF-β1 (MA1-21595, mouse, Invitrogen), β-ACTIN (A5441, mouse, Sigma-Aldrich), and GAPDH (sc-47724, mouse, Santa Cruz) primary antibodies at dilutions of 1:1,000 each. We carried out detection using ECL (Amersham Biosciences).

Bromodeoxyuridine proliferation assay

We detected HCF proliferation *in situ* using BrdU as previously described [13]. Briefly, we labeled HCFs with BrdU for 16 hours and then fixed the cells with ethanol and immunostained them for BrdU incorporation using the BrdU-Labeling and Detection kit II (Sigma-Aldrich) according to the manufacturer's recommendations. We determined the total number of nuclei by manual counting of DAPI-stained nuclei in 6 random fields per coverslip (original magnification, X20). We counted all BrdU-positive nuclei on each coverslip. We acquired digital photographs of fluorescence with a Keyence microscope (BZ-X810) and processed them with Adobe Photoshop.

Wound migration assay

We detected HCF migration as previously described [13]. Briefly, we plated 1×10^4 HCFs in each well of a 2-well Culture-Insert 35-mm μ-Dish (81176, Ibidi, Fitchburg, WI). Once the cells were confluent and high-cell density/confluency led to contact inhibition of proliferation, we removed the silicone insert on each dish to reveal a defined cell-free gap. We replaced the medium and took images at 0 and 24 hours. Subsequently, we quantified the distance between

cell fronts in three wells of each group using ImageJ software. We measured the initial open (cell-free) areas (0 hour) to serve as the total open area, and we calculated the percentage of open area after 24 hours to determine the migratory potential of HCFs.

Supplementary References

1. Bayoumi AS, Teoh JP, Aonuma T, Yuan Z, Ruan X, Tang Y, et al. MicroRNA-532 protects the heart in acute myocardial infarction, and represses prss23, a positive regulator of endothelial-to-mesenchymal transition. Cardiovasc Res. 2017; 113: 1603-14.
2. Bayoumi AS, Park KM, Wang Y, Teoh JP, Aonuma T, Tang Y, et al. A carvedilol-responsive microRNA, miR-125b-5p protects the heart from acute myocardial infarction by repressing pro-apoptotic bak1 and klf13 in cardiomyocytes. J Mol Cell Cardiol. 2017; 114: 72-82.
3. Tang Y, Wang Y, Park KM, Hu Q, Teoh JP, Broskova Z, et al. MicroRNA-150 protects the mouse heart from ischaemic injury by regulating cell death. Cardiovasc Res. 2015; 106: 387-97.
4. Ramakrishna S, Kim IM, Petrovic V, Malin D, Wang IC, Kalin TV, et al. Myocardium defects and ventricular hypoplasia in mice homozygous null for the Forkhead Box M1 transcription factor. Dev Dyn. 2007; 236: 1000-13.
5. Kim IM, Ackerson T, Ramakrishna S, Tretiakova M, Wang IC, Kalin TV, et al. The Forkhead Box m1 transcription factor stimulates the proliferation of tumor cells during development of lung cancer. Cancer Res. 2006; 66: 2153-61.
6. Aonuma T, Moukette B, Kawaguchi S, Barupala NP, Sepulveda MN, Corr C, et al. Cardiomyocyte microRNA-150 confers cardiac protection and directly represses proapoptotic small proline-rich protein 1A. JCI Insight. 2021; 6: e150405.
7. Kim IM, Tilley DG, Chen J, Salazar NC, Whalen EJ, Violin JD, et al. Beta-blockers alprenolol and carvedilol stimulate beta-arrestin-mediated EGFR transactivation. Proc Natl Acad Sci U S A. 2008; 105: 14555-60.
8. Park KM, Teoh JP, Wang Y, Broskova Z, Bayoumi AS, Tang Y, et al. Carvedilol-responsive microRNAs, miR-199a-3p and -214 protect cardiomyocytes from simulated ischemia-reperfusion injury. Am J Physiol Heart Circ Physiol. 2016; 311: H371-83.
9. Aurora AB, Mahmoud AI, Luo X, Johnson BA, van Rooij E, Matsuzaki S, et al. MicroRNA-214 protects the mouse heart from ischemic injury by controlling Ca(2)(+) overload and cell death. J Clin Invest. 2012; 122: 1222-32.
10. Teoh JP, Bayoumi AS, Aonuma T, Xu Y, Johnson JA, Su H, et al. Beta-arrestin-biased agonism of beta-adrenergic receptor regulates Dicer-mediated microRNA maturation to promote cardioprotective signaling. J Mol Cell Cardiol. 2018; 118: 225-36.
11. Kim IM, Ramakrishna S, Gusarova GA, Yoder HM, Costa RH, Kalinichenko VV. The forkhead box m1 transcription factor is essential for embryonic development of pulmonary vasculature. J Biol Chem. 2005; 280: 22278-86.
12. Kim IM, Wolf MJ, Rockman HA. Gene deletion screen for cardiomyopathy in adult Drosophila identifies a new notch ligand. Circ Res. 2010; 106: 1233-43.
13. Aonuma T, Moukette B, Kawaguchi S, Barupala NP, Sepulveda MN, Frick K, et al. MiR-150 attenuates maladaptive cardiac remodeling mediated by long noncoding RNA MIAT and directly represses profibrotic Hoxa4. Circ Heart Fail. 2022; 15: e008686.

Supplementary Table 1. Echocardiographic parameters in anesthetized WT, miR-150 KO, or miR-150 KO;*Sprr1a*$^{hypo/hypo}$ mice before they were randomly assigned to 6 experimental groups.

Week 0	WT		miR-150 KO		miR-150 KO;*Sprr1a*$^{hypo/hypo}$	
	Sham (n=18)	MI (n=21)	Sham (n=18)	MI (n=19)	Sham (n=19)	MI (n=20)
CO (ml/min)	18.81 ± 0.60	18.91 ± 0.23	19.43 ± 0.29	19.59 ± 0.28	19.10 ± 0.24	19.35 ± 0.28
EF (%)	68.36 ± 0.51	68.41 ± 0.60	69.94 ± 0.74	69.87 ± 0.67	68.50 ± 0.57	70.20 ± 0.68
FS (%)	37.51 ± 0.40	37.44 ± 0.47	38.67 ± 0.60	38.63 ± 0.54	37.52 ± 0.45	38.89 ± 0.55
HR (bpm)	518 ± 8.1	526 ± 4.99	537 ± 3.92	529 ± 5.86	525 ± 4.38	526± 5.37
SV (μl)	36.35 ± 1.10	35.99 ± 0.48	36.21 ± 0.43	37.07 ± 0.50	36.43 ± 0.45	36.81 ± 0.47
End volume, diastole (μl)	53.17 ± 1.58	52.70 ± 0.95	51.82 ± 0.55	53.06 ± 0.53	53.20 ± 0.56	52.46 ± 0.60
End volume, systole (μl)	16.82 ± 0.59	16.71 ± 0.56	15.60 ± 0.48	15.99 ± 0.39	16.77 ± 0.37	15.65 ± 0.44
LVAW, diastole (mm)	0.64 ± 0.02	0.64 ± 0.02	0.62 ± 0.01	0.64 ± 0.01	0.62 ± 0.01	0.62 ± 0.01
LVAW, systole (mm)	0.98 ± 0.02	0.98 ± 0.02	1.01 ± 0.01	1.01 ± 0.01	1.01 ± 0.01	1.00 ± 0.01
LVID, diastole (mm)	3.56 ± 0.05	3.54 ± 0.03	3.53 ± 0.02	3.56 ± 0.01	3.56 ± 0.02	3.54 ± 0.02
LVID, systole (mm)	2.23 ± 0.03	2.22 ± 0.03	2.16 ± 0.03	2.19 ± 0.02	2.23 ± 0.02	2.16 ± 0.02
LVPW, diastole (mm)	0.62 ± 0.01	0.66 ± 0.02	0.62 ± 0.01	0.62 ± 0.01	0.63 ± 0.01	0.66 ± 0.02
LVPW, systole (mm)	0.97 ± 0.02	0.95 ± 0.02	0.98 ± 0.01	0.98 ± 0.01	0.98 ± 0.01	0.98 ± 0.01

Abbreviations: MI = myocardial infarction, CO = cardiac output, EF = ejection fraction, FS = fractional shortening, HR = heart rate, SV = stroke volume, LVAW = left ventricular anterior wall thickness, LVID = left ventricular internal diameter, and LVPW = left ventricular posterior wall thickness. All values are expressed as mean ± SEM.

Supplementary Table 2. Echocardiographic parameters in anesthetized WT, miR-150 KO, or miR-150 KO;*Sprr1a*$^{hypo/hypo}$ mice at 3 days after Sham or MI surgery.

3 days post-surgery	WT		miR-150 KO		miR-150 KO;*Sprr1a*$^{hypo/hypo}$	
	Sham (n=12)	MI (n=10)	Sham (n=18)	MI (n=19)	Sham (n=19)	MI (n=20)
CO (ml/min)	19.12 ± 0.44	16.97 ± 0.53**	19.64 ± 0.35	15.99 ± 0.35***	19.02 ± 0.38	17.52 ± 0.31**§§
EF (%)	68.29 ± 0.84	49.12 ± 0.92***	68.86 ± 0.58	44.80 ± 0.73***##	68.78 ± 0.65	53.45 ± 1.25***§§
FS (%)	37.20 ± 0.67	24.43 ± 0.55***	37.78 ± 0.46	21.90 ± 0.41***##	37.71 ± 0.52	27.14 ± 0.77***§§
HR (bpm)	527 ± 8.95	528 ± 10.24	543 ± 4.05	525 ± 5.83*	535 ± 5.07	536 ± 4.71
SV (µl)	36.27 ± 0.46	32.17 ± 0.86***	36.15 ± 0.47	30.49 ± 0.62***	35.52 ± 0.53	32.71 ± 0.53***§
End volume, diastole (µl)	53.17 ± 0.56	65.55 ± 1.56***	52.52 ± 0.66	68.32 ± 1.56***	51.64 ± 0.56	61.54 ± 1.00**§§
End volume, systole (µl)	16.90 ± 0.47	33.38 ± 1.14***	16.38 ± 0.41	37.83 ± 1.19***#	16.12 ± 0.36	28.83 ± 1.20***§§
LVAW, diastole (mm)	0.62 ± 0.02	0.63 ± 0.03	0.61 ± 0.01	0.58 ± 0.01*#	0.63 ± 0.01	0.61 ± 0.01§
LVAW, systole (mm)	1.00 ± 0.01	0.93 ± 0.02*	1.01 ± 0.01	0.87 ± 0.02***	1.01 ± 0.01	0.92 ± 0.02**
LVID, diastole (mm)	3.56 ± 0.02	3.90 ± 0.04***	3.55 ± 0.02	3.95 ± 0.04***	3.52 ± 0.02	3.79 ± 0.03***§§§
LVID, systole (mm)	2.23 ± 0.03	2.95 ± 0.04***	2.21 ± 0.02	3.11 ± 0.05***#	2.19 ± 0.02	2.76 ± 0.05***§§§
LVPW, diastole (mm)	0.64 ± 0.02	0.62 ± 0.01	0.63 ± 0.01	0.62 ± 0.01	0.64 ± 0.01	0.62 ± 0.01
LVPW, systole (mm)	0.99 ± 0.02	1.00 ± 0.01	0.96 ± 0.01	0.92 ± 0.02#	0.98 ± 0.01	0.96 ± 0.01

Abbreviations: MI = myocardial infarction, CO = cardiac output, EF = ejection fraction, FS = fractional shortening, HR = heart rate, SV = stroke volume, LVAW = left ventricular anterior wall thickness, LVID = left ventricular internal diameter, and LVPW = left ventricular posterior wall thickness. All values are expressed as mean ± SEM. $^{*}P$<0.05, $^{**}P$<0.01, or $^{***}P$<0.001 vs. sham within same group. $^{\#}P$<0.05 or $^{\#\#}P$<0.01 vs. WT MI. $^{\S}P$<0.05, $^{\S\S}P$<0.01, or $^{\S\S\S}P$<0.001 vs. miR-150 KO MI. Only parameters, which were statistically significant between groups, are highlighted with red fonts.

Supplementary Table 3. Echocardiographic parameters in anesthetized WT, miR-150 KO, or miR-150 KO;*Sprr1a*$^{hypo/hypo}$ mice at 4 weeks after Sham or MI surgery.

4 weeks post-surgery	WT		miR-150 KO		miR-150 KO;*Sprr1a*$^{hypo/hypo}$	
	Sham (n=18)	MI (n=18)	Sham (n=18)	MI (n=18)	Sham (n=18)	MI (n=18)
CO (ml/min)	19.68 ± 0.35	15.02 ± 0.33***	19.57 ± 0.34	16.21 ± 0.52***	19.62 ± 0.27	17.08 ± 0.36***
EF (%)	68.77 ± 0.77	41.18 ± 0.82***	68.33 ± 0.58	38.23 ± 0.87***#	68.72 ± 0.65	48.83 ± 1.04***§§§
FS (%)	37.76 ± 0.61	19.84 ± 0.45***	37.39 ± 0.46	18.34 ± 0.47***#	37.70 ± 0.52	24.32 ± 0.62***§§§
HR (bpm)	542 ± 8.56	534 ± 8.76	536 ± 4.46	511 ± 7.33**	540 ± 2.89	514 ± 6.05***
SV (µl)	36.36 ± 0.51	28.24 ± 0.72***	36.53 ± 0.58	31.75 ± 0.97***##	36.36 ± 0.47	33.23 ± 0.59***
End volume, diastole (µl)	52.90 ± 0.65	68.99 ± 2.16***	53.48 ± 0.77	83.51 ± 2.59***###	52.92 ± 0.54	68.43 ± 1.52***§§§
End volume, systole (µl)	16.54 ± 0.51	40.75 ± 1.78***	16.94 ± 0.42	51.76 ± 2.02***###	16.56 ± 0.40	35.20 ± 1.38***§§§
LVAW, diastole (mm)	0.66 ± 0.01	0.59 ± 0.02**	0.63 ± 0.01	0.59 ± 0.01**	0.64 ± 0.01	0.63 ± 0.01§
LVAW, systole (mm)	1.01 ± 0.01	0.77 ± 0.02***	1.01 ± 0.01	0.80 ± 0.02***	1.01 ± 0.01	0.94 ± 0.02**§§§
LVID, diastole (mm)	3.56 ± 0.02	3.97 ± 0.05***	3.58 ± 0.02	4.30 ± 0.06***###	3.56 ± 0.02	3.96 ± 0.04***§§§
LVID, systole (mm)	2.21 ± 0.03	3.18 ± 0.05***	2.24 ± 0.02	3.51 ± 0.06***###	2.22 ± 0.02	3.00 ± 0.05***§§§
LVPW, diastole (mm)	0.65 ± 0.01	0.68 ± 0.02	0.64 ± 0.01	0.62 ± 0.01##	0.65 ± 0.01	0.66 ± 0.01§
LVPW, systole (mm)	0.98 ± 0.01	0.97 ± 0.02	0.99 ± 0.01	0.88 ± 0.02***##	0.97 ± 0.01§	0.94 ± 0.01§

Abbreviations: MI = myocardial infarction, CO = cardiac output, EF = ejection fraction, FS = fractional shortening, HR = heart rate, SV = stroke volume, LVAW = left ventricular anterior wall thickness, LVID = left ventricular internal diameter, and LVPW = left ventricular posterior wall thickness. All values are expressed as mean ± SEM. $^{*}P$<0.05, $^{**}P$<0.01, or $^{***}P$<0.001 vs. sham within same group. $^{\#}P$<0.05, $^{\#\#}P$<0.01, or $^{\#\#\#}P$<0.001 vs. WT MI. $^{\S}P$<0.05, $^{\S\S}P$<0.01, or $^{\S\S\S}P$<0.001 vs. miR-150 KO MI. Only parameters, which were statistically significant between groups, are highlighted with red fonts.

Supplementary Table 4. Echocardiographic parameters in anesthetized WT, miR-150 KO, or miR-150 KO;*Sprr1a*$^{hypo/hypo}$ mice at 8 weeks after Sham or MI surgery.

8 weeks post-surgery	WT		miR-150 KO		miR-150 KO;*Sprr1a*$^{hypo/hypo}$	
Echocardiography	Sham (n=18)	MI (n=18)	Sham (n=18)	MI (n=18)	Sham (n=18)	MI (n=18)
CO (ml/min)	19.17 ± 0.46	14.86 ± 0.61***	19.71 ± 0.35	14.86 ± 0.59***	19.70 ± 0.38	17.88 ± 0.48**§§§
EF (%)	67.80 ± 0.64	38.77 ± 0.57***	68.54 ± 0.51	31.92 ± 1.43***###	69.19 ± 0.76	46.86 ± 1.03***§§§
FS (%)	36.94 ± 0.50	18.56 ± 0.31***	37.55 ± 0.41	15.07 ± 0.72***###	38.10 ± 0.62	23.19 ± 0.59***§§§
HR (bpm)	527± 8.50	501 ± 10.27	534 ± 4.87	496 ± 5.20***	534 ± 3.63	520 ± 5.13*§§
SV (μl)	36.36 ± 0.61	29.64 ± 1.07***	36.92 ± 0.48	29.95 ± 1.06***	36.89 ± 0.62	34.34 ± 0.70*§§
End volume, diastole (μl)	53.64 ± 0.58	76.65 ± 2.93***	53.86 ± 0.53	96.78 ± 5.06***##	53.31 ± 0.60	73.95 ± 2.34***§§§
End volume, systole (μl)	17.28 ± 0.37	47.01 ± 1.98***	16.94 ± 0.31	66.83 ± 4.84***###	16.41 ± 0.42	39.62 ± 1.95***§§§
LVAW, diastole (mm)	0.67 ± 0.02	0.60 ± 0.02*	0.63 ± 0.01	0.54 ± 0.01***#	0.67 ± 0.02	0.61 ± 0.01*§§
LVAW, systole (mm)	1.02 ± 0.02	0.72 ± 0.03***	1.02 ± 0.01	0.74 ± 0.03***	1.03 ± 0.01	0.90 ± 0.02***§§§
LVID, diastole (mm)	3.58 ± 0.02	4.15 ± 0.06***	3.58 ± 0.01	4.57 ± 0.10***##	3.57 ± 0.02	4.09 ± 0.05***§§§
LVID, systole (mm)	2.25 ± 0.02	3.38 ± 0.06***	2.24 ± 0.02	3.89 ± 0.11***###	2.21 ± 0.02	3.14 ± 0.06***§§§
LVPW, diastole (mm)	0.67 ± 0.02	0.70 ± 0.02	0.65 ± 0.01	0.62 ± 0.01###	0.65 ± 0.01	0.66 ± 0.01
LVPW, systole (mm)	0.99 ± 0.01	0.94 ± 0.01*	0.99 ± 0.01	0.89 ± 0.02***#	0.97 ± 0.01	0.95 ± 0.01§§
Morphometric data	Sham (n=13)	MI (n=12)	Sham (n=12)	MI (n=12)	Sham (n=12)	MI (n=12)
HW/BW (mg/g)	4.12 ± 0.10	5.28 ± 0.21***	4.16 ± 0.12	5.47 ± 0.17***	3.97 ± 0.06	4.43 ± 0.12**§§§
LVW/BW (mg/g)	3.12 ± 0.06	4.08 ± 0.15***	3.00 ± 0.13	4.19 ± 0.12***	3.04 ± 0.06	3.46 ± 0.10**§§§

Abbreviations: MI = myocardial infarction, CO = cardiac output, EF = ejection fraction, FS = fractional shortening, HR = heart rate, SV = stroke volume, LVAW = left ventricular anterior wall thickness, LVID = left ventricular internal diameter, LVPW = left ventricular posterior wall thickness, HW = heart weight, BW = body weight, and LVW = left ventricular weight. All values are expressed as mean ± SEM. $^{*}P<0.05$, $^{**}P<0.01$, or $^{***}P<0.001$ vs. sham within same group. $^{\#}P<0.05$, $^{\#\#}P<0.01$, or $^{\#\#\#}P<0.001$ vs. WT MI. $^{\S\S}P<0.01$ or $^{\S\S\S}P<0.001$ vs. miR-150 KO MI. Only parameters, which were statistically significant between groups, are highlighted with red fonts.

Supplementary Figure 1

1 Sham WT
2 Sham miR-150 KO
3 Sham miR-150 KO;*Sprr1a*$^{hypo/hypo}$
4 MI WT
5 MI miR-150 KO
6 MI miR-150 KO;*Sprr1a*$^{hypo/hypo}$

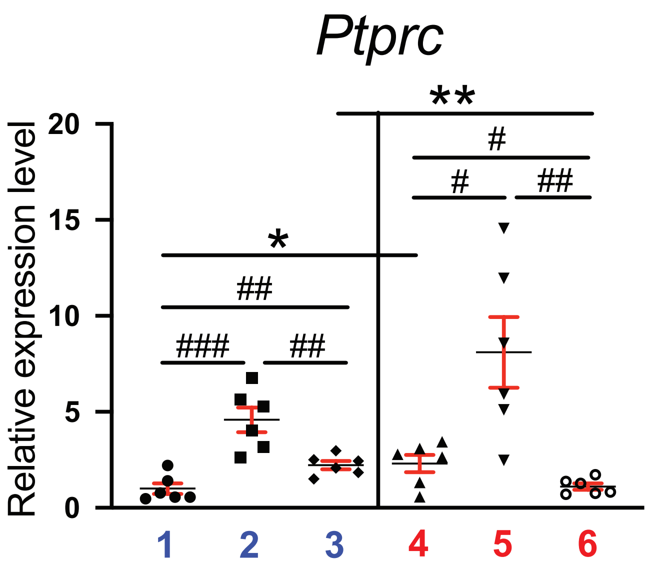

Supplementary Figure 1. MiR-150 KO hearts exhibit increased expression of proinflammatory *Ptprc*, which is attenuated by *Sprr1a* knockdown. qRT-PCR analyses of *Ptprc* in the ischemic area from WT, miR-150 KO, and miR-150 KO;$Sprr1a^{hypo/hypo}$ mouse hearts at 8 weeks post-MI. Data are represented as the fold change in gene expression normalized to *Gapdh*. Two-way ANOVA with Tukey's multiple comparison test. *$P<0.05$ or **$P<0.01$ vs. sham for each genotype; $^{\#}P<0.05$, $^{\#\#}P<0.01$, or $^{\#\#\#}P<0.001$ vs. WT or miR-150 KO. Data are presented as the mean ± SEM.

Supplementary Figure 2

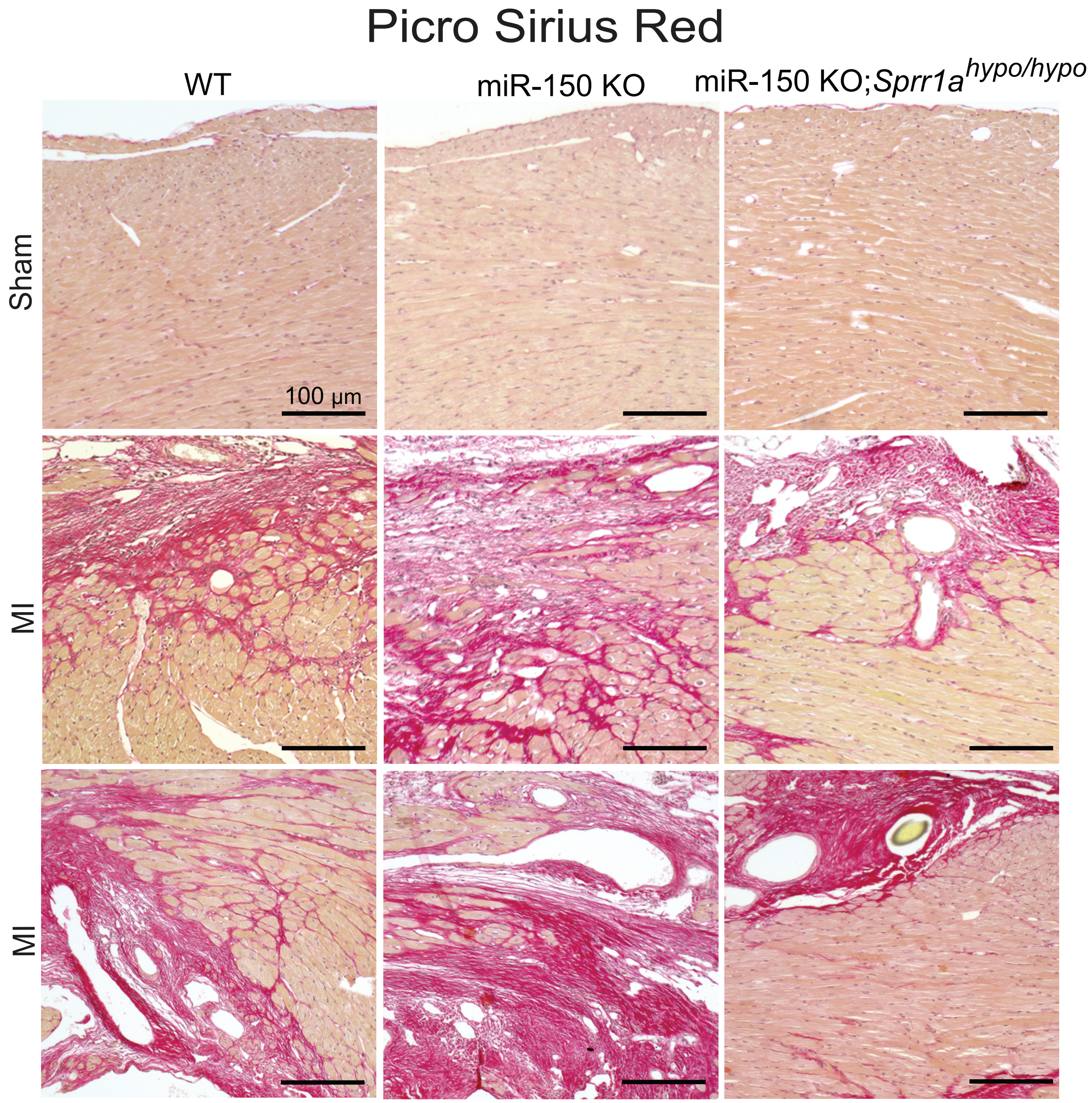

Supplementary Figure 2. MiR-150 deletion increases cardiac fibrosis post-MI, which is reversed by *Sprr1a* knockdown. Representative picrosirius red zooming images from heart sections in 6 experimental groups at 8 weeks post-MI. The middle and bottom zooming images are from heart sections of the ischemic area and border zone in 3 experimental groups at 8 weeks post-MI. Scale bars: 100 µm.

Supplementary Figure 3

1 Sham WT **2** Sham miR-150 KO **3** Sham miR-150 KO;*Sprr1a*$^{hypo/hypo}$

4 MI WT **5** MI miR-150 KO **6** MI miR-150 KO;*Sprr1a*$^{hypo/hypo}$

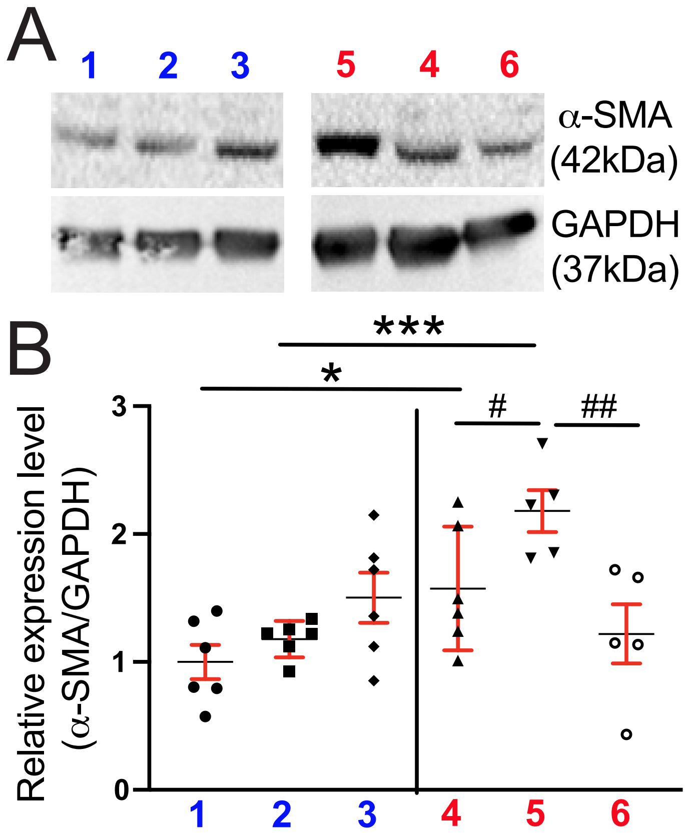

Supplementary Figure 3. *Sprr1a* is a significant functional target for miR-150 repression on fibrotic α-SMA in mouse hearts. α-SMA protein levels were measured in ischemic areas from WT, miR-150 KO, and miR-150 KO;*Sprr1a*$^{hypo/hypo}$ mouse hearts at 8 weeks post-MI. Representative blot images in **A** were cropped from noncontiguous lanes in the same blot that was run on the same gel, and the images are separated by white spaces (See **unedited original blot images in Online Supporting Data**). N=5–6 per group. Two-way ANOVA with Tukey's multiple comparison test. **P*<0.05 or ****P*<0.001 vs. sham for each genotype; $^{\#}P<0.05$ or $^{\#\#}P<0.01$ vs. WT or miR-150 KO. Data are presented as the mean ± SEM.

Supplementary Figure 4

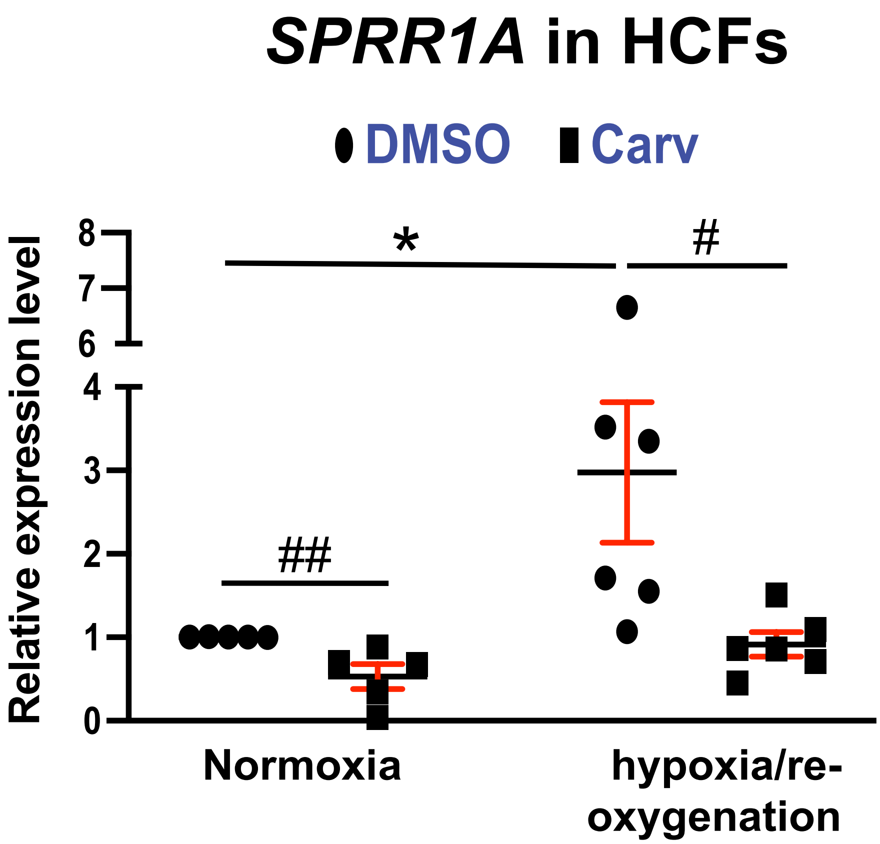

Supplementary Figure 4. *SPRR1A* is downregulated in human cardiac fibroblasts by carvedilol. Human primary CFs (HCFs) were treated with 1 μM Carv for 16 h and subjected to either normoxia (basal) or hypoxia/reoxygenation. qRT-PCR analyses for *SPRR1A* were then performed. Carv inhibits the expression of *SPRR1A* in HCFs subjected to hypoxia/reoxygenation. Moreover, *SPRR1A* is upregulated in HCFs after hypoxia/reoxygenation. Data are shown as the fold induction of expression normalized to *GAPDH*. Two-way ANOVA with Tukey's multiple comparison test. *$P<0.05$ vs. normoxia. $^{\#}P<0.05$ or $^{\#\#}P<0.01$ vs. DMSO. Data are presented as the mean ± SEM.

Supplemantary Figure 5

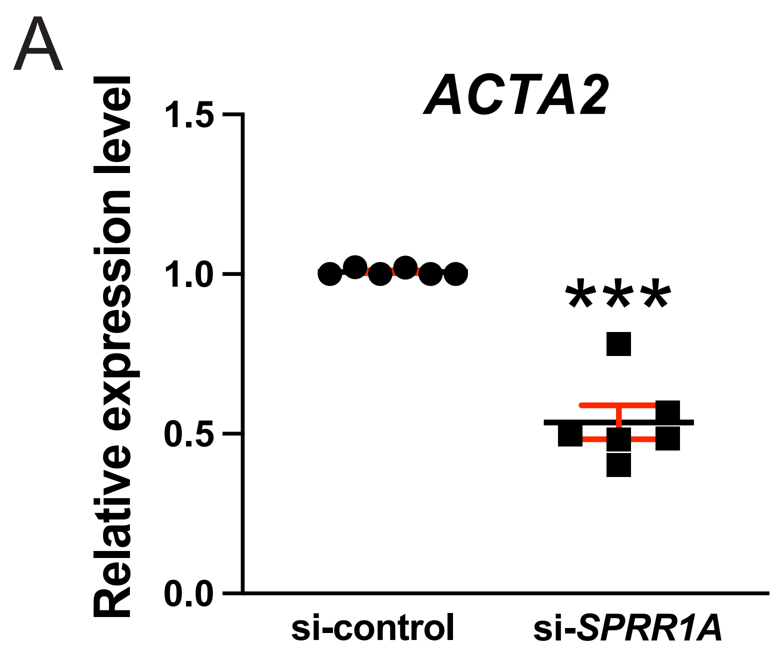

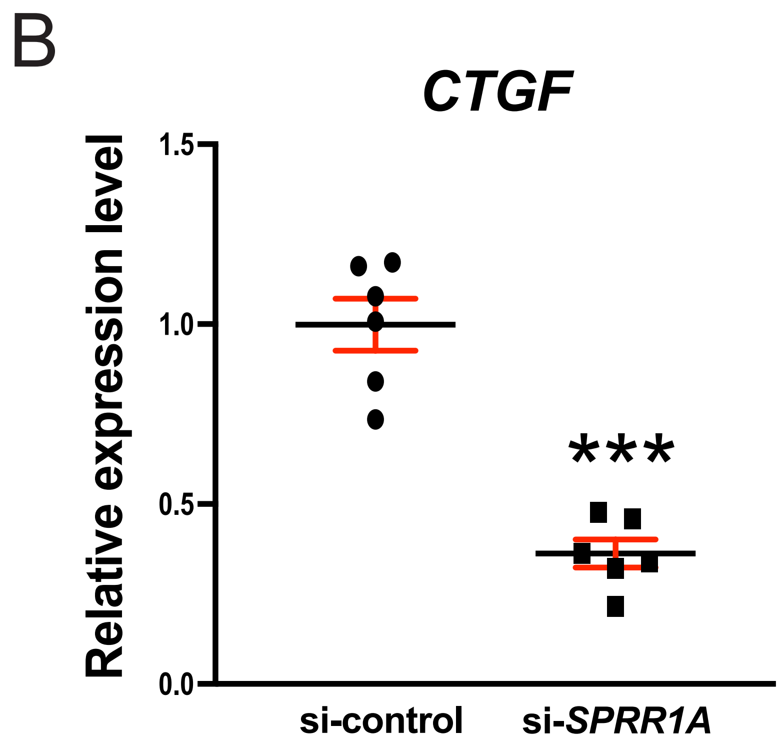

Supplementary Figure 5. Knockdown of *SPRR1A* in human cardiac fibroblasts reduces the expression of profibrotic *ACTA2* and *CTGF*. A and B, Human primary CFs (HCFs) were transfected with control scramble siRNA (si-control) or *SPRR1A* siRNA (si-*SPRR1A*). qRT-PCR analyses of *ACTA2* (**A**) and *CTGF* (**B**) were then performed. N=6 per group. Data are shown as the fold induction of gene expression normalized to *GAPDH*. Unpaired 2-tailed t-test. ****P*<0.001 vs. si-control. Data are presented as the mean ± SEM.

Supplemantary Figure 6

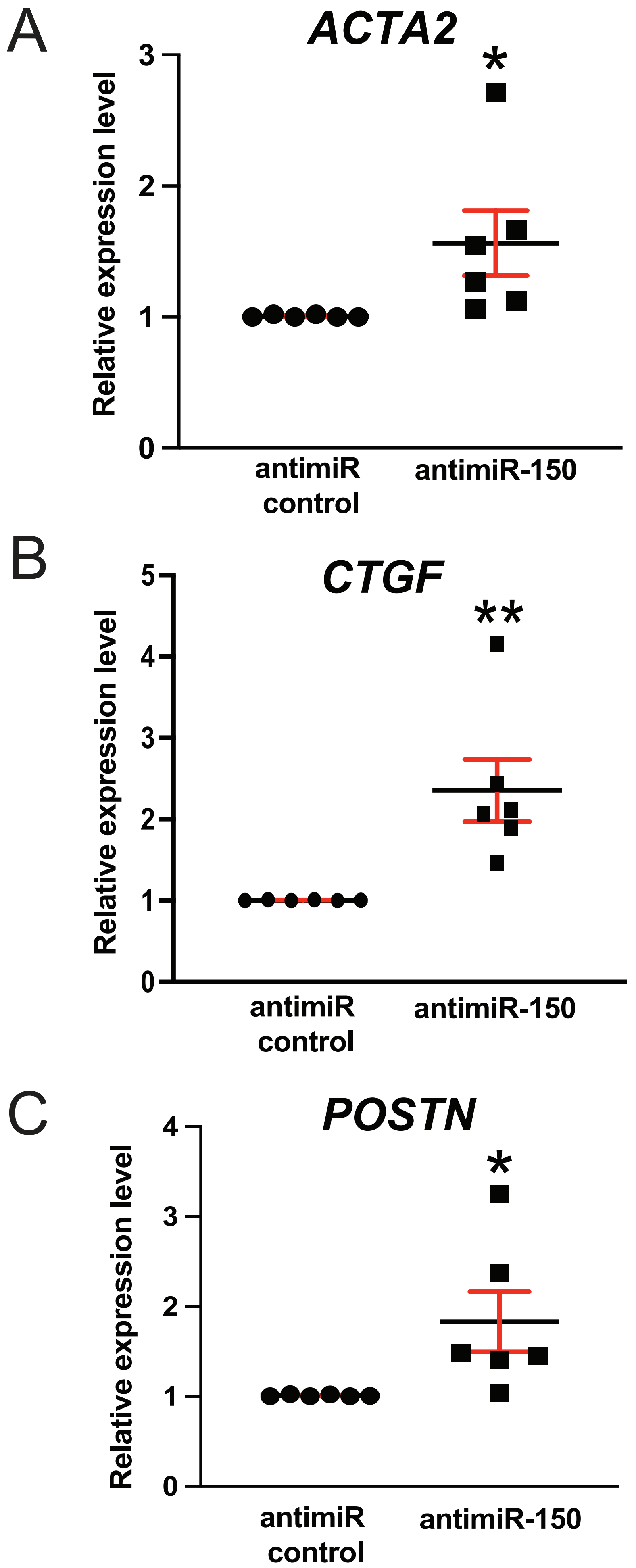

Supplementary Figure 6. MiR-150 knockdown increases the expression of *ACTA2, CTGF*, and *POSTN*. A–C, Human primary CFs (HCFs) were transfected with antimiR control or antimiR-150. qRT-PCR analyses of *ACTA2* (**A**), *CTGF* (**B**), and *POSTN* (**C**) were then performed. N=6 per group. Data are shown as the fold induction of gene expression normalized to *GAPDH*. Unpaired 2-tailed t-test. **P*<0.05 or ***P*<0.01 vs. antimiR control. Data are presented as the mean ± SEM.

Supplementary Figure 7

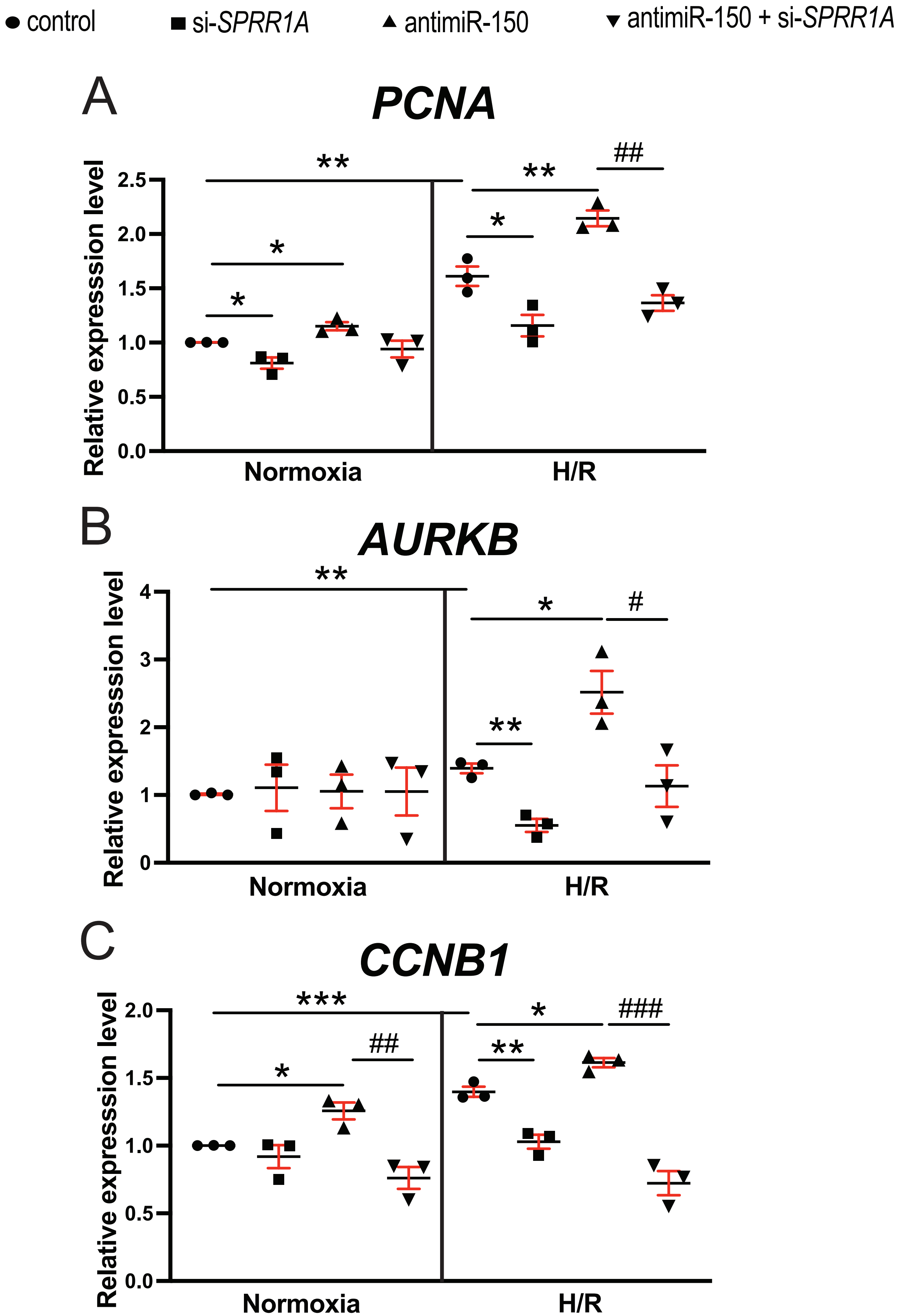

Supplementary Figure 7. *SPRR1A* is necessary for miR-150-dependent regulation of cell proliferation markers, *PCNA*, *AURKB*, and *CCNB1* in HCFs. RNA interference with *SPRR1A* reverses the increased expression of cell proliferation markers, *PCNA*, *AURKB*, and *CCNB1* mediated by antimiR-150 in HCFs. HCFs were transfected as indicated and subjected to normoxia or hypoxia/reoxygenation (H/R). qRT-PCR analyses for *PCNA* (**A**), *AURKB* (**B**), or *CCNB1* (**C**) were then performed to check their expression after the indicated transfection. Data were normalized to *GAPDH* and expressed relative to controls. N=3 per group. One-way ANOVA with Tukey's multiple comparison test. **P*<0.05, ***P*<0.01, or ****P*<0.001 vs. control: either si-control or antimiR control. $^{\#}P<0.05$, $^{\#\#}P<0.01$ or $^{\#\#\#}P<0.001$ vs. antimiR-150. Data are presented as the mean ± SEM.

Supplementary Figure 8

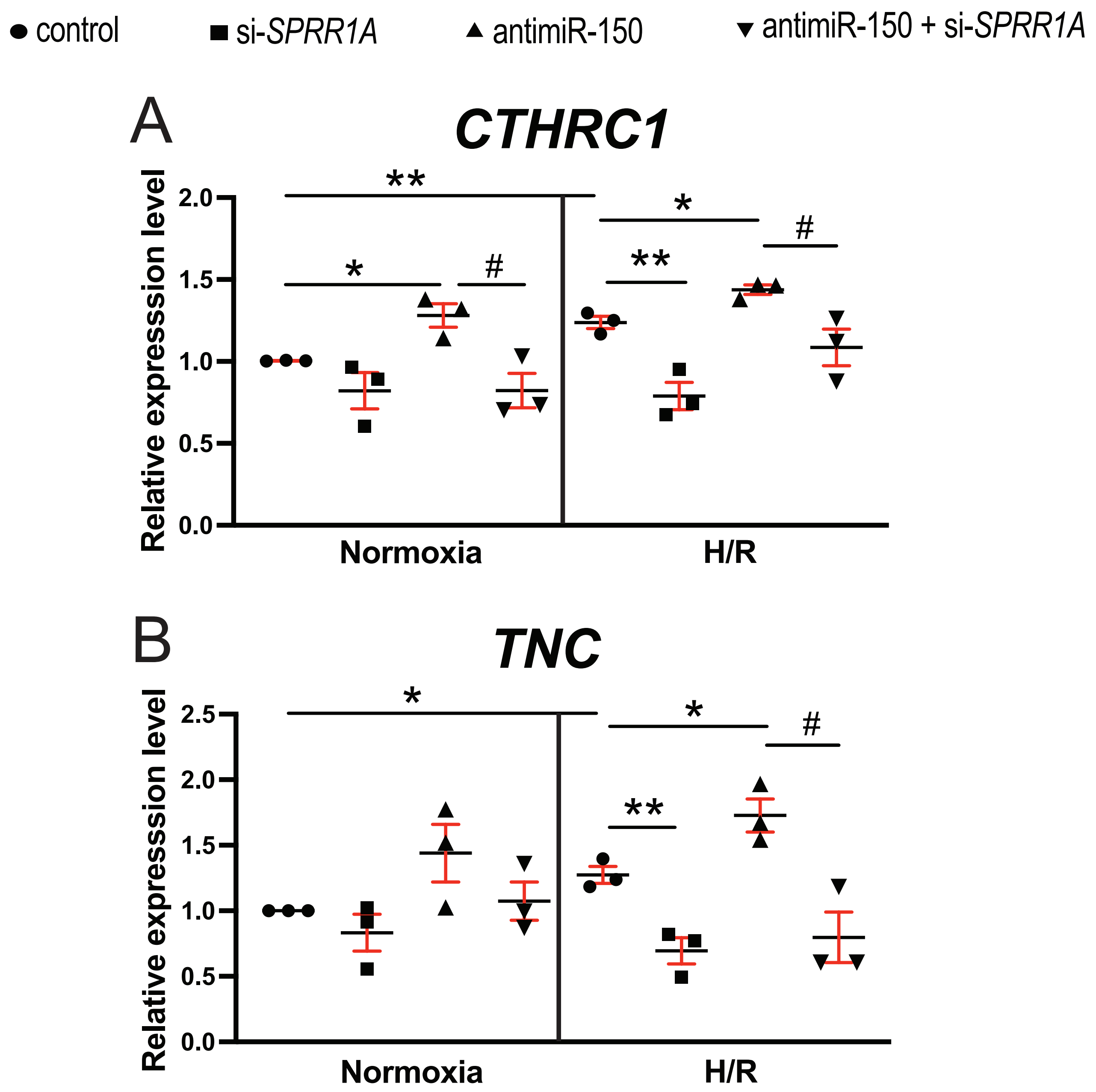

Supplementary Figure 8. *SPRR1A* is necessary for miR-150-dependent regulation of cell migration markers, *CTHRC1* and *TNC* in HCFs. RNA interference with *SPRR1A* reverses the increased expression of migration markers, *CTHRC1* and *TNC* mediated by antimiR-150 in HCFs. HCFs were transfected as indicated and subjected to normoxia or hypoxia/reoxygenation (H/R). qRT-PCR analyses for *CTHRC1* (**A**) or *TNC* (**B**) were then performed to check their expression after the indicated transfection. Data were normalized to *GAPDH* and expressed relative to controls. N=3 per group. One-way ANOVA with Tukey's multiple comparison test. *P<0.05 or **P<0.01 vs. control: either si-control or antimiR control. $^{\#}P$<0.05 vs. antimiR-150. Data are presented as the mean ± SEM.

Supplementary Figure 9

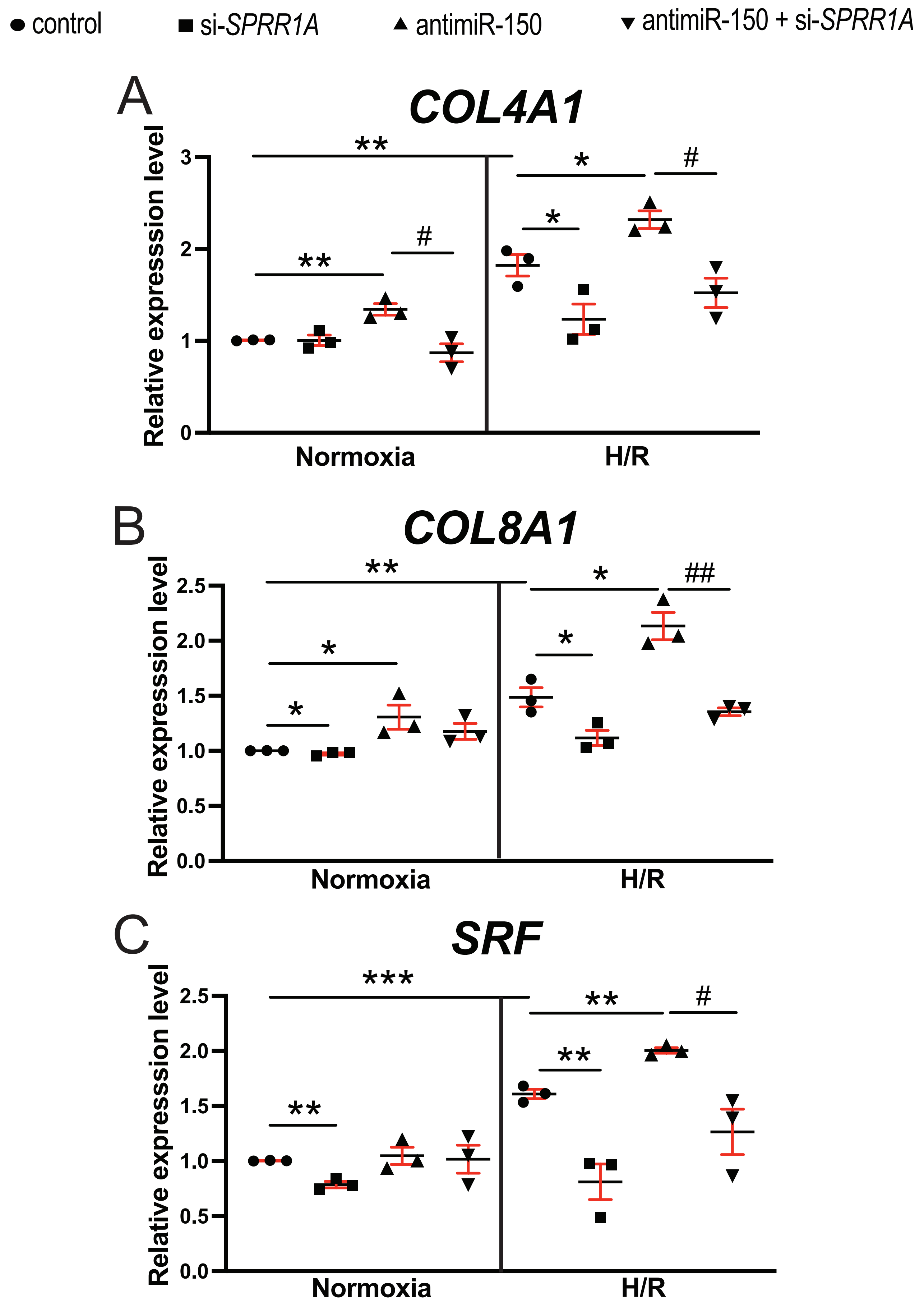

Supplementary Figure 9. *SPRR1A* is necessary for miR-150-dependent regulation of profibrotic *COL4A1*, *COL8A1,* and *SRF*. RNA interference with *SPRR1A* reverses the increased expression of profibrotic *COL4A1*, *COL8A1,* and *SRF* mediated by antimiR-150 in HCFs. HCFs were transfected as indicated and subjected to normoxia or hypoxia/reoxygenation (H/R). qRT-PCR analyses for *COL4A1* (**A**), *COL8A1* (**B**) or *SRF* (**C**) were then performed to check their expression after the indicated transfection. Data were normalized to *GAPDH* and expressed relative to controls. N=3 per group. One-way ANOVA with Tukey's multiple comparison test. *$P<0.05$, **$P<0.01$, or ***$P<0.001$ vs. control: either si-control or antimiR control. $^{\#}P<0.05$ or $^{\#\#}P<0.01$ vs. antimiR-150. Data are presented as the mean ± SEM.

Supplementary Figure 10

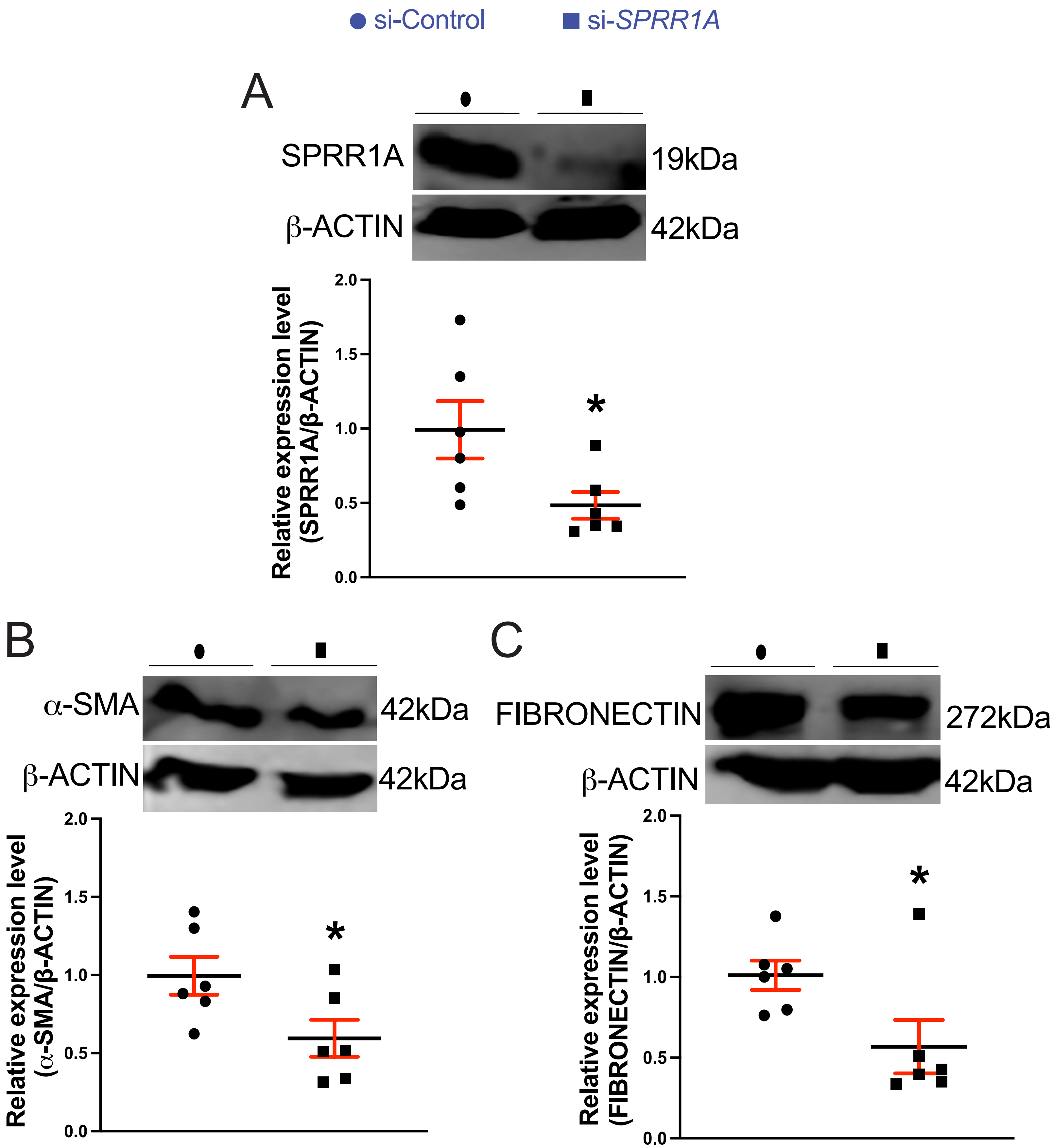

Supplementary Figure 10. Knockdown of *SPRR1A* in human cardiac fibroblasts reduces the levels of the profibrotic markers: α-SMA and FIBRONECTIN. A–C, Human primary CFs (HCFs) were transfected with control scramble siRNA (si-control) or *SPRR1A* siRNA (si-*SPRR1A*). Western blotting analyses of SPRR1A (**A**), α-SMA (**B**), and FIBRONECTIN (**C**) were then performed. N=6 per group. Data are shown as the fold induction of protein levels normalized to β-ACTIN. Unpaired 2-tailed t-test. **P*<0.05 vs. si-control. Data are presented as the mean ± SEM.

Supplementary Figure 11

● control ■ si-*SPRR1A* ▲ antimiR-150 ▼ antimiR-150 + si-*SPRR1A*

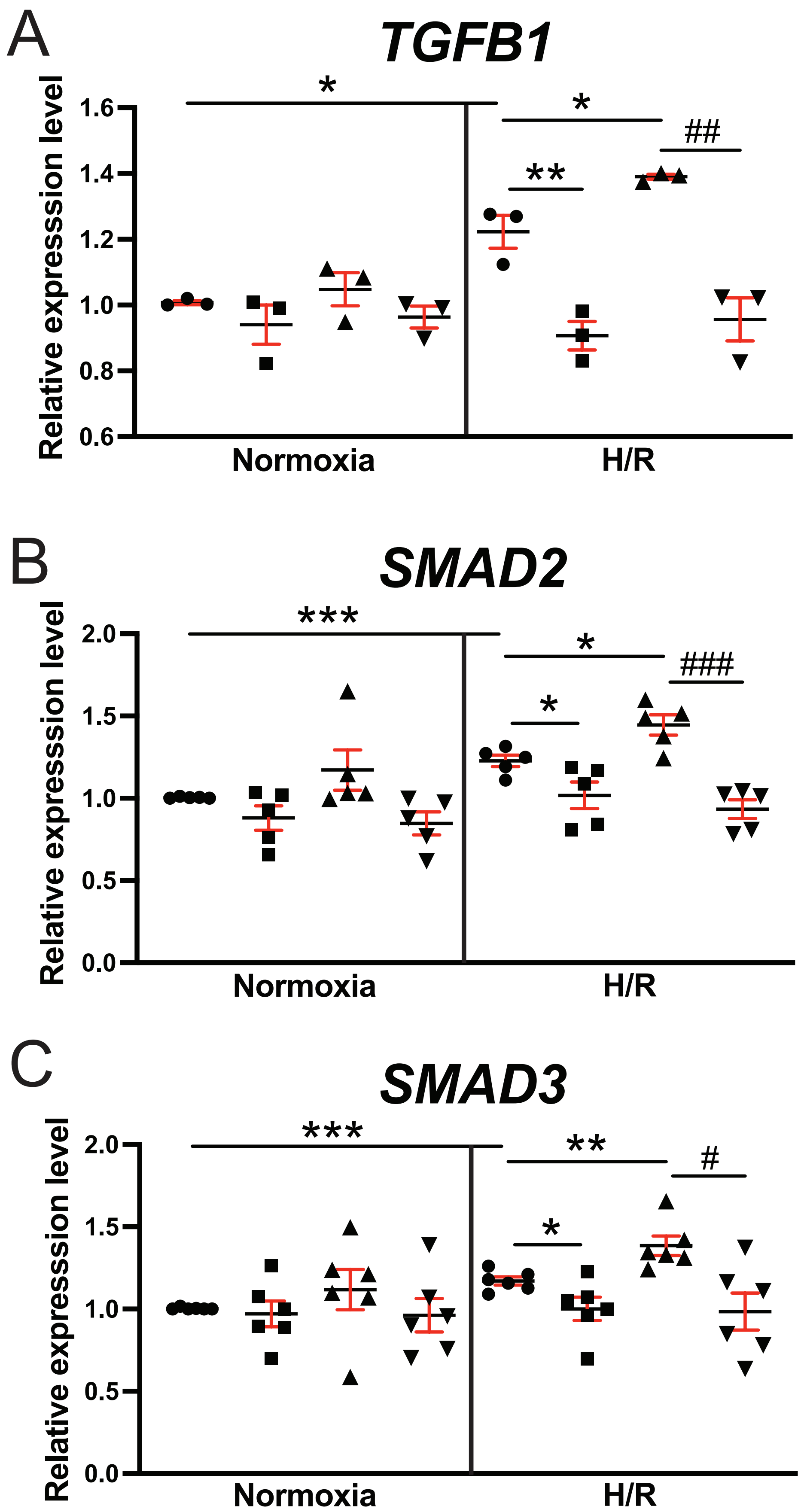

Supplementary Figure 11. *SPRR1A* is necessary for miR-150-dependent regulation of profibrotic *TGFB1*, *SMAD2*, and *SMAD3*. RNA interference with *SPRR1A* reverses the increased expression of profibrotic *TGFB1*, *SMAD2*, and *SMAD3* mediated by antimiR-150 in HCFs. HCFs were transfected as indicated and subjected to normoxia or hypoxia/reoxygenation (H/R). qRT-PCR analyses for *TGFB1* (**A**), *SMAD2* (**B**), or *SMAD3* (**C**) were then performed to check their expression after the indicated transfection. Data were normalized to *GAPDH* and expressed relative to controls. N=3–6 per group. One-way ANOVA with Tukey's multiple comparison test. $^{*}P<0.05$, $^{**}P<0.01$, or $^{***}P<0.001$ vs. control: either si-control or antimiR control. $^{\#}P<0.05$, $^{\#\#}P<0.01$, or $^{\#\#\#}P<0.001$ vs. antimiR-150. Data are presented as the mean ± SEM.

Supplementary Figure 12

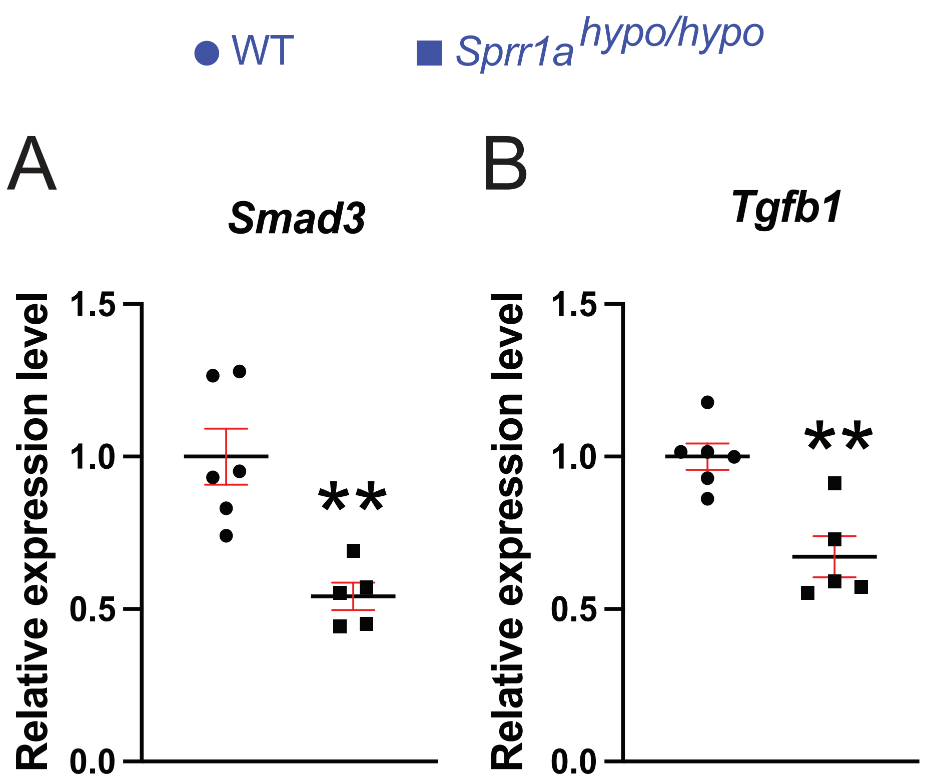

Supplementary Figure 12. Knockdown of *Sprr1a* in mice reduces the cardiac levels of *Smad3* and *Tgfb1*. qRT-PCR analysis of profibrotic *Smad3* (**A**) or *Tgfb1* (**B**) expression in WT and $Sprr1a^{hypo/hypo}$ mouse left ventricles. Data are shown as the fold induction of gene expression normalized to *Gapdh*. N=5 or 6 per group. Unpaired 2-tailed t-test. $^{**}P<0.01$ vs. WT. Data are presented as the mean ± SEM.

Supplementary Figure 13

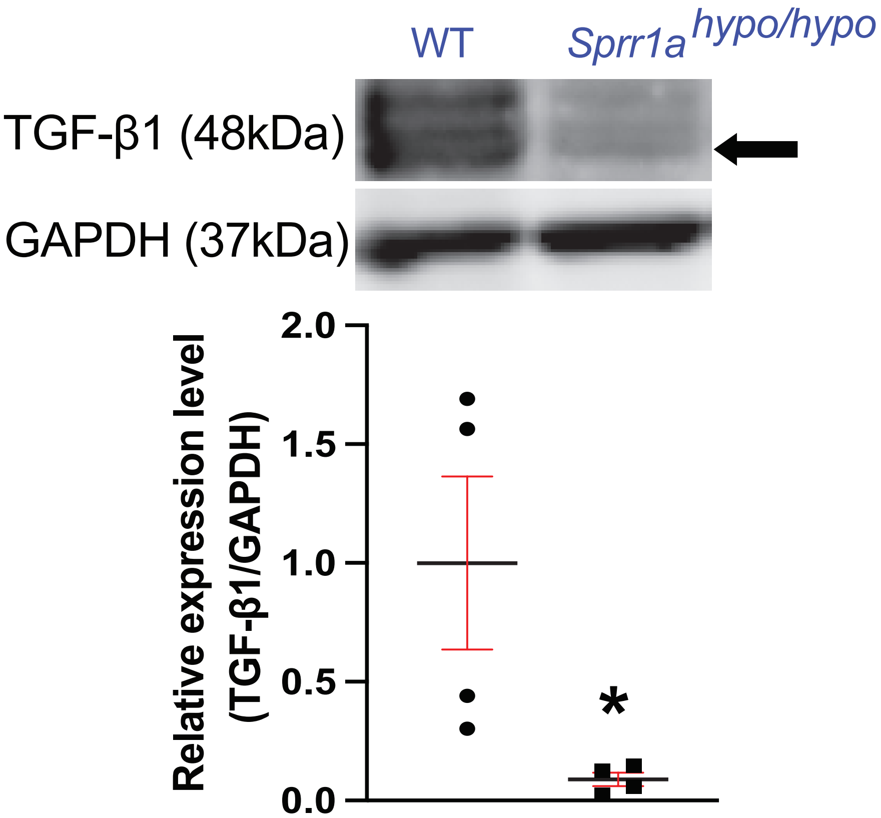

Supplementary Figure 13. Knockdown of *Sprr1a* in mice reduces the cardiac levels of TGF-β1. TGF-β1 protein levels were measured in WT and *Sprr1a*$^{hypo/hypo}$ mouse left ventricles. N=4 per group. Unpaired 2-tailed t-test. *P<0.05 vs. WT. Data are presented as the mean ± SEM.

Supplementary Figure 14

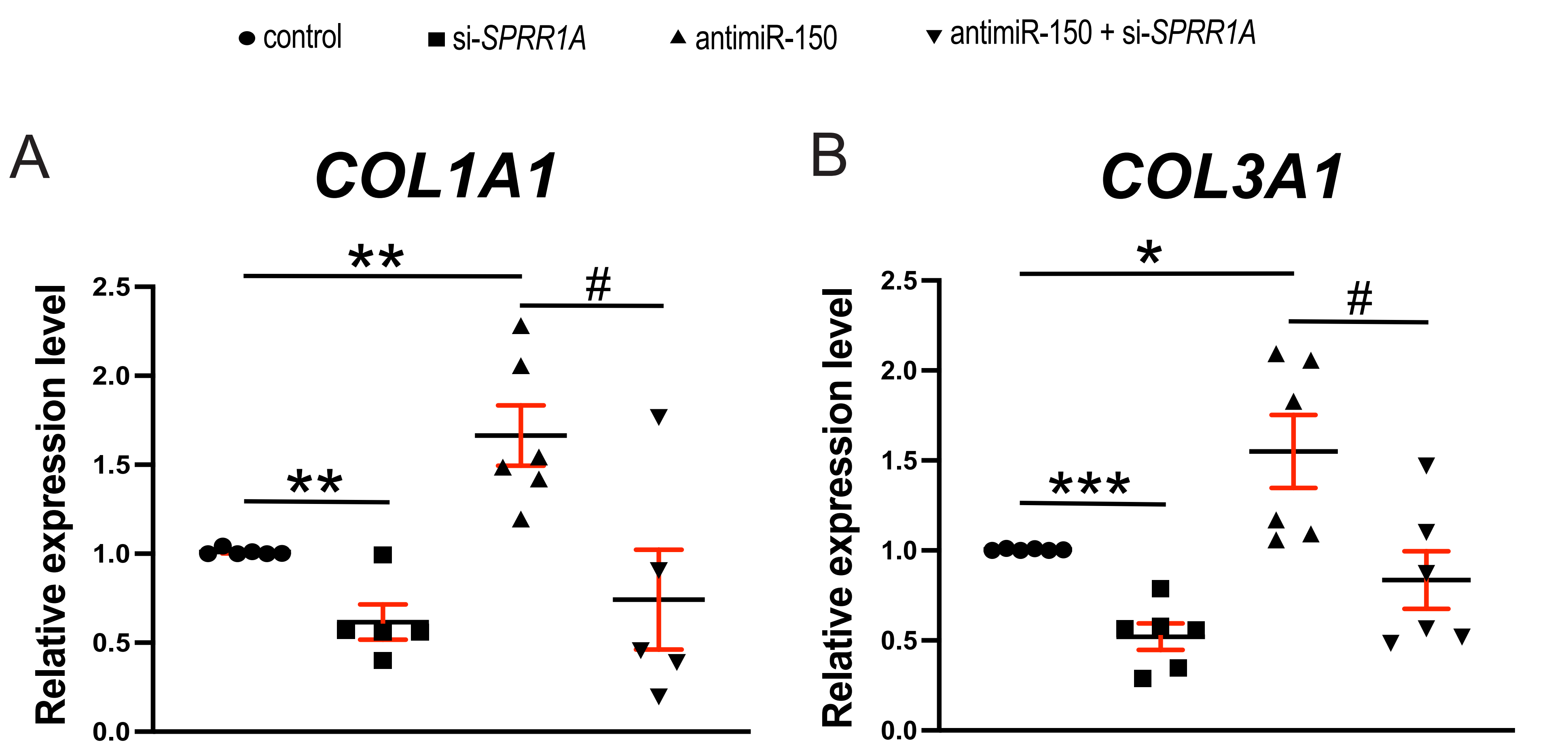

Supplementary Figure 14. *SPRR1A* is necessary for miR-150-dependent regulation of profibrotic *COL1A1* and *COL3A1*. A and B, RNA interference with *SPRR1A* reverses the increased expression of profibrotic *COL1A1* and *COL3A1* mediated by antimiR-150 in HCFs. HCFs were transfected as indicated, and qRT-PCR analyses for *COL1A1* (**A**) or *COL3A1* (**B**) were then performed to check their expression after the indicated transfection. Data were normalized to *GAPDH* and expressed relative to controls. N=6 per group. One-way ANOVA with Tukey's multiple comparison test. *P<0.05, **P<0.01, or ***P<0.001 vs. control: either si-control or antimiR control. $^{\#}P$<0.05 vs. antimiR-150. Data are presented as the mean ± SEM.
