# Supplementary material for: SPRR1A is a key downstream effector of MiR-150 during both maladaptive cardiac remodeling in mice and human cardiac fibroblast activation
Source: Cell Death Dis. 2023 Jul 19;14(7):446. doi: 10.1038/s41419-023-05982-y (PMC10356860; doi:10.1038/s41419-023-05982-y)

Figure 6D

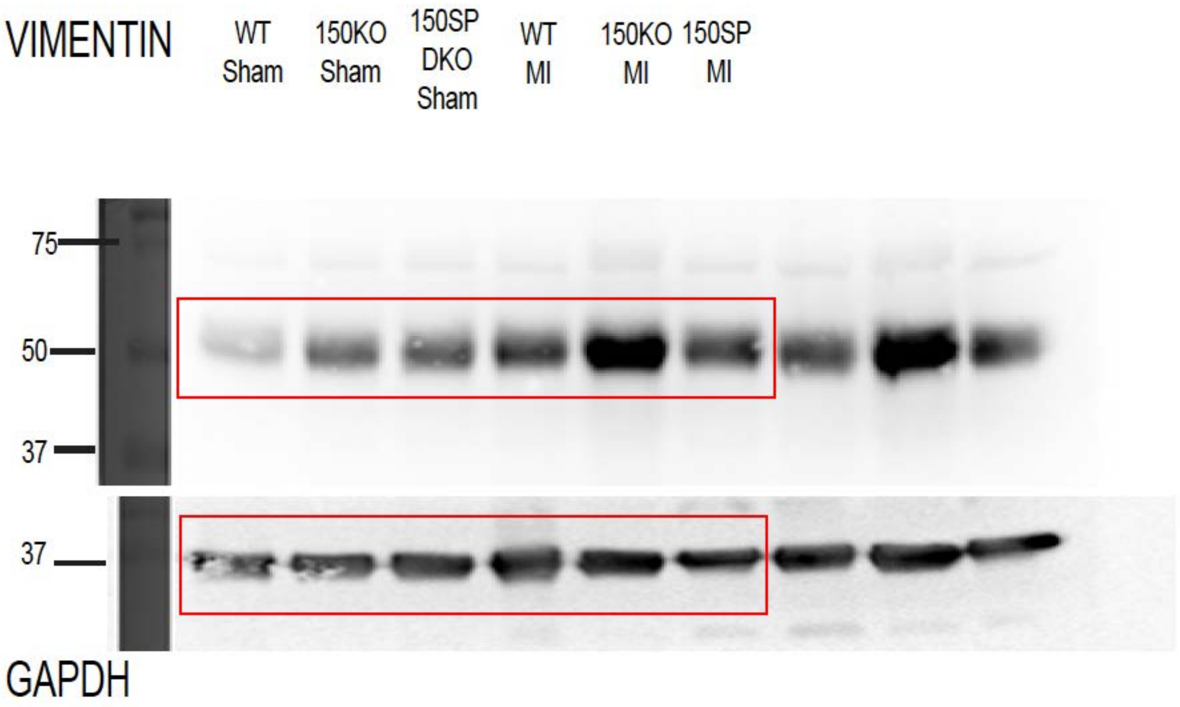

VIMENTIN
WT Sham
150KO Sham
150SP DKO Sham
WT MI
150KO MI
150SP MI
75
50
37
37
GAPDH

Supplementary Figure 3A

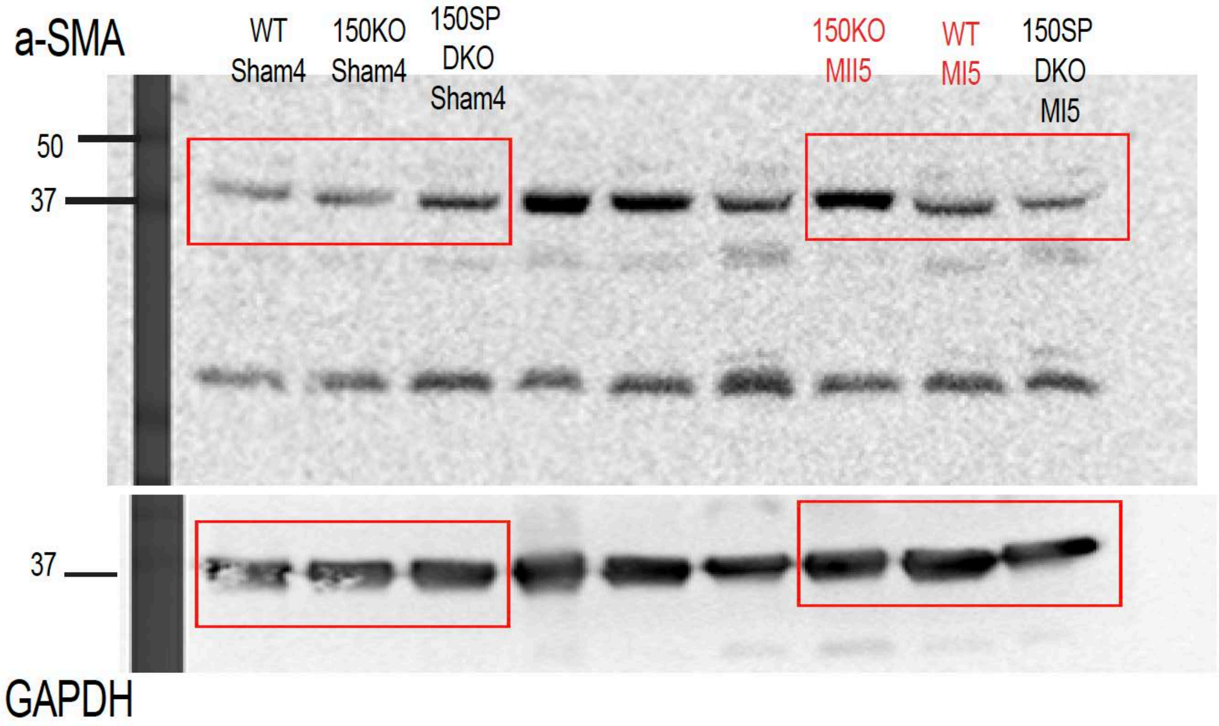

Supplementary Figure 10A

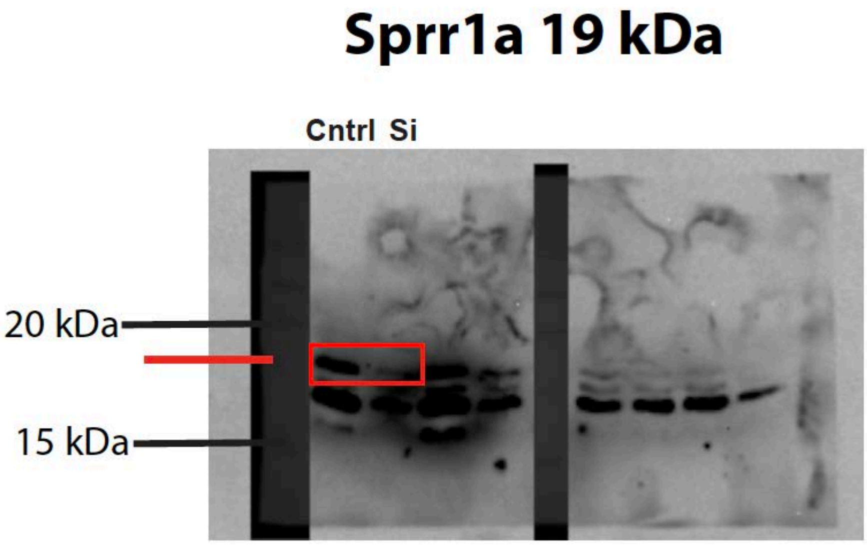

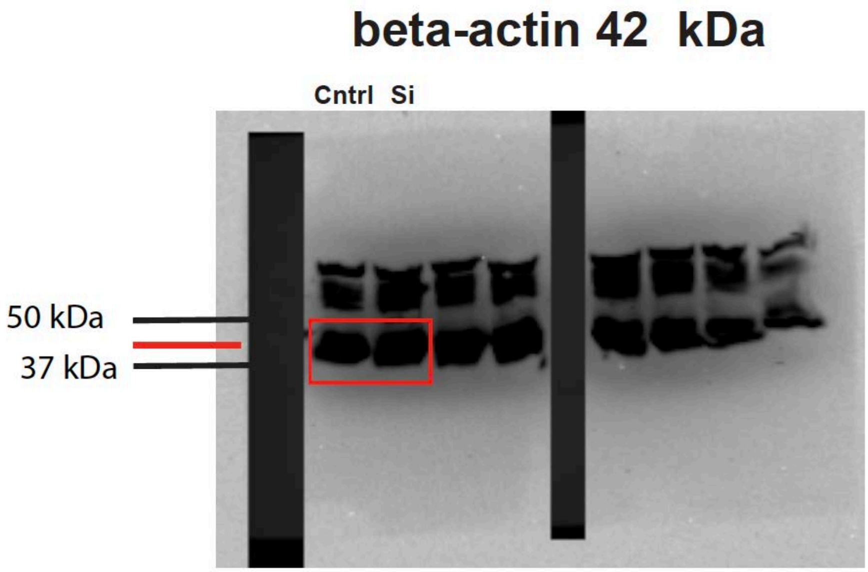

Supplementary Figure 10B

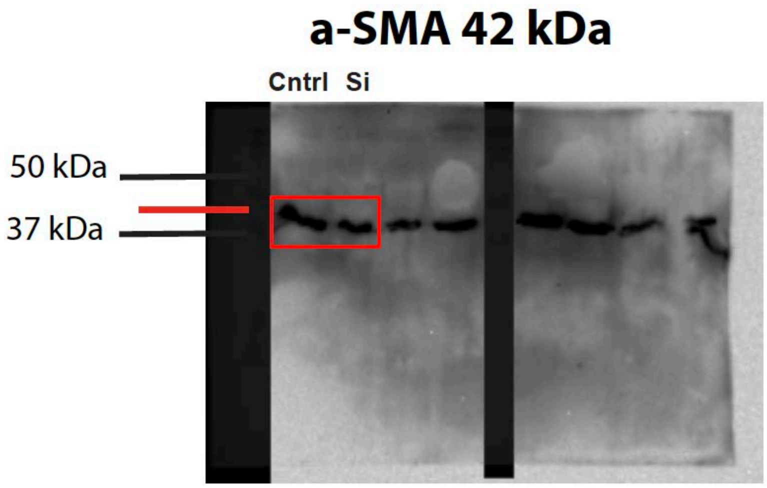

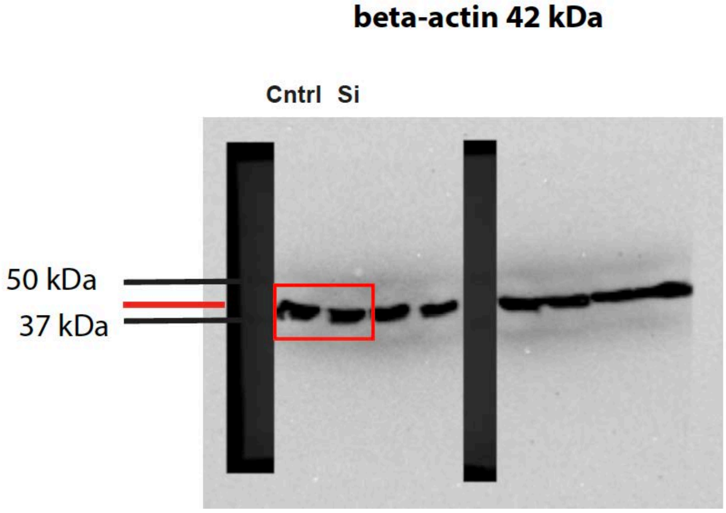

Supplementary Figure 10C

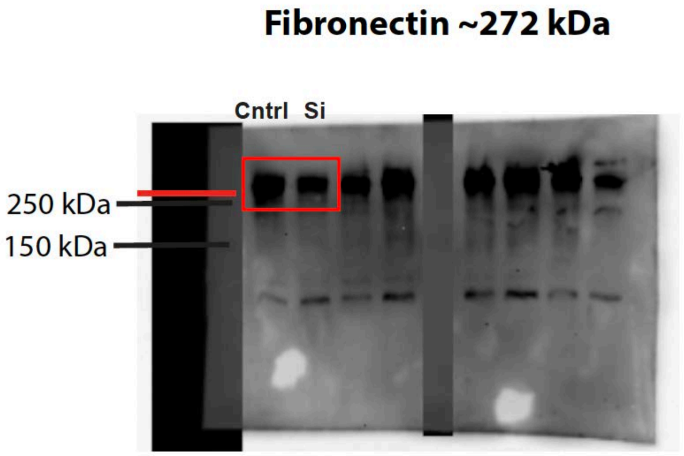

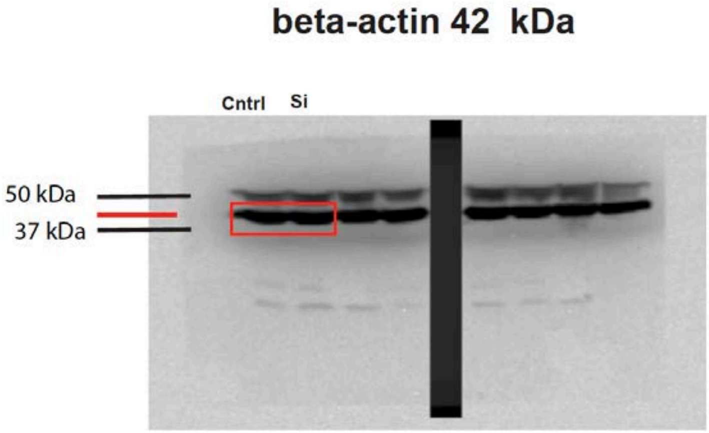

Supplementary Figure 13

TGF-beta1

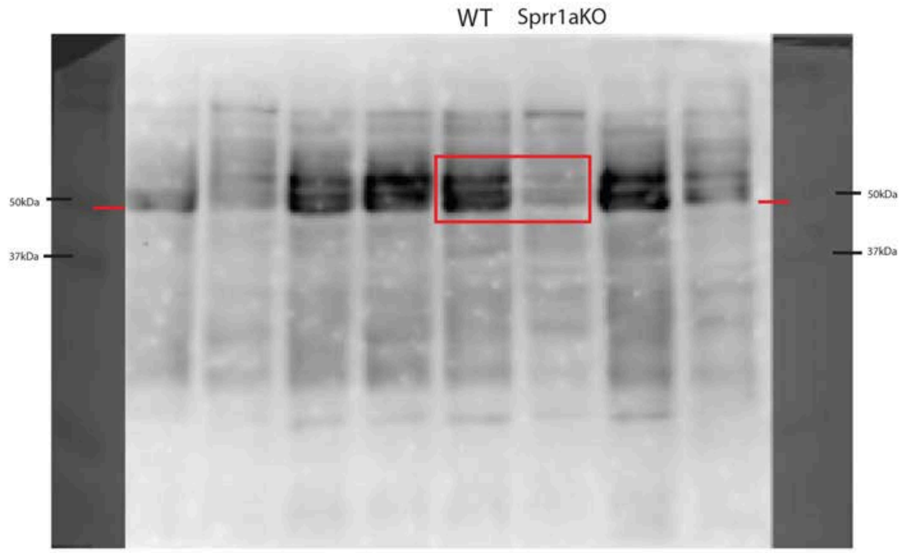

GAPDH

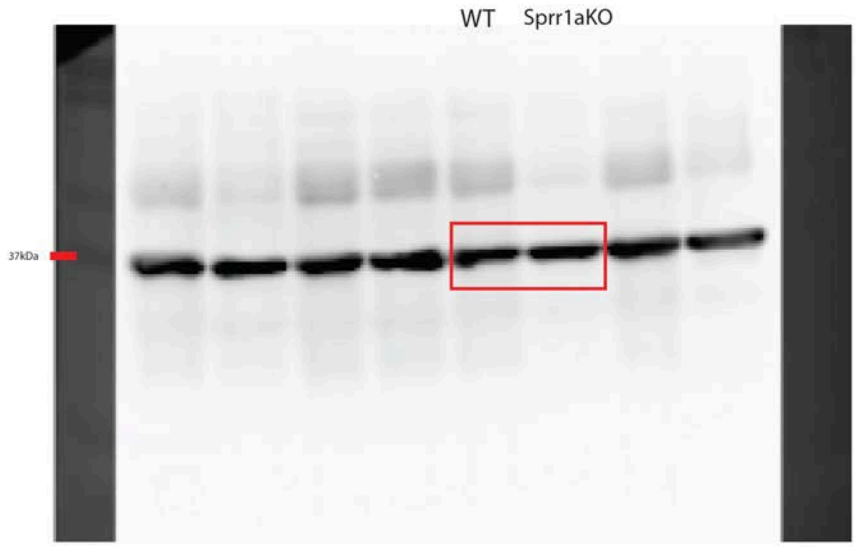

Supplement: Supplementary file 3 — Full and uncropped western blots [file 41419_2023_5982_MOESM3_ESM.pdf]
